# Comparative Phylogenomics of Pathogenic and Nonpathogenic Species

**DOI:** 10.1534/g3.115.022806

**Published:** 2015-11-25

**Authors:** Emily Whiston, John W. Taylor

**Affiliations:** Department of Plant and Microbial Biology, University of California, Berkeley, California 94720

**Keywords:** fungal evolution, genomics, coccidioides, onygenales, phylogenetics

## Abstract

The Ascomycete Onygenales order embraces a diverse group of mammalian pathogens, including the yeast-forming dimorphic fungal pathogens *Histoplasma capsulatum*, *Paracoccidioides* spp. and *Blastomyces dermatitidis*, the dermatophytes *Microsporum* spp. and *Trichopyton* spp., the spherule-forming dimorphic fungal pathogens in the genus *Coccidioides*, and many nonpathogens. Although genomes for all of the aforementioned pathogenic species are available, only one nonpathogen had been sequenced. Here, we enhance comparative phylogenomics in Onygenales by adding genomes for *Amauroascus mutatus*, *Amauroascus niger*, *Byssoonygena ceratinophila*, and *Chrysosporium queenslandicum*—four nonpathogenic Onygenales species, all of which are more closely related to *Coccidioides* spp. than any other known Onygenales species. Phylogenomic detection of gene family expansion and contraction can provide clues to fungal function but is sensitive to taxon sampling. By adding additional nonpathogens, we show that LysM domain-containing proteins, previously thought to be expanding in some Onygenales, are contracting in the *Coccidioides-Uncinocarpus* clade, as are the self-nonself recognition Het loci. The denser genome sampling presented here highlights nearly 800 genes unique to *Coccidiodes*, which have significantly fewer known protein domains and show increased expression in the endosporulating spherule, the parasitic phase unique to *Coccidioides* spp. These genomes provide insight to gene family expansion/contraction and patterns of individual gene gain/loss in this diverse order—both major drivers of evolutionary change. Our results suggest that gene family expansion/contraction can lead to adaptive radiations that create taxonomic orders, while individual gene gain/loss likely plays a more significant role in branch-specific phenotypic changes that lead to adaptation for species or genera.

Comparative genomic analysis has identified new sources of genetic variation, most importantly gene family expansion/contraction, itself a consequence of individual gene duplication and gene loss. This source of genetic variation complements the well-known generation of variation by nucleotide substitution and DNA rearrangement, and another source, horizontal gene transfer, whose prevalence among closely related organisms has been revealed by comparative genomics. Discovery of gene family expansion/contraction and associated gene gain/loss depends on the density of taxon sampling and the quality of the genome assemblies and annotations (Demuth and Hahn 2009). In fungi, the Ascomycota order Onygenales has been a model for such studies because it has a diverse range of human pathogenic fungi as well as nonpathogens. The Onygenales pathogens with sequenced genomes include the yeast-forming dimorphic fungal pathogens *Histoplasma capsulatum*, *Paracoccidioides* spp. and *Blastomyces dermatitidis*, the dermatophytes *Microsporum* spp. and *Trichopyton* spp., and the spherule-forming dimorphic fungal pathogens *Coccidioides immitis* and *Coccidioides posadasii* (Martinez *et al.* 2012; Sharpton *et al.* 2009). In the Onygenales, both phylogenomic (Sharpton *et al.* 2009) and population genomics comparisons (Neafsey *et al.* 2010) have found evidence of gene gain and gene loss, gene family contractions and expansions, and horizontal gene transfer in the form of introgression between sister *Coccidioides* species.

The disease coccidioidomycosis (colloquially known as Valley Fever or San Joaquin Valley Fever) and its causative agents, *Coccidioides* (*Co*.) *immitis* and *Co. posadasii*, are found in the Central Valley of California, and throughout the American Southwest, as well as in Mexico, parts of Central America, and South America (Fisher *et al.* 2002; Pfaller and Diekema 2010; Taylor and Fisher 2003). Coccidioidomycosis affects a diverse range of mammals, including humans, and is potentially fatal even in immunocompetent adults (Hector *et al.* 2011). In the environment, *Coccidioides* grows as a typical mycelium, and produces clonal arthroconidia—the infectious agents. When arthroconidia enter the host’s lungs, they undergo a morphological switch to become endosporulating spherules, a growth form unique to *Coccidioides* spp. among all known fungi. The morphological switch occurs when conidia, ca. 1–3 × 3–6 µm in diameter, enlarge to form spherule initials, which then grow isotopically to > 60 μm in diameter (Borchers and Gershwin 2010). Nuclei divide within mature spherules and are packaged into hundreds of endospores. Mature spherules continue to expand until they rupture, releasing endospores, which then enlarge to continue the spherule growth cycle; endospores can enter the bloodstream and disseminate to almost any soft tissue, where they may cause serious disease (Cox and Magee 2004). Additional parasitic growth forms have been described from coccidioidomycosis patients: pleomorphic hyphae cells and fungal balls (formed by septate hyphae) (Hagman *et al.* 2000; Munoz-Hernandez *et al.* 2014).

Broad phylogenomic studies in the Onygenales have provided a better understanding of their biology; for example, gene family contractions in cellulases and other plant metabolism genes, and gene family expansions in proteases, keratinases and other animal tissue metabolism genes, indicate that these fungi switched from a nutrition based on plants to one based on animals (Desjardins *et al.* 2011; Martinez *et al.* 2012; Sharpton *et al.* 2009). Similar patterns have been observed in the Hypocreales, another order of Ascomycota that harbors animal pathogens and nonpathogens, indicating that the switch from plant to animal substrates has evolved independently in these two orders (Muszewska *et al.* 2011; Sharpton *et al.* 2009). In the Onygenales, this hypothesis has been tested experimentally; in *Uncinocarpus reesii*, hyphal growth was restricted on carbohydrates and considerably improved on proteins (Desjardins *et al.* 2011). In addition to these major order-wide observations, branch-specific gene family changes have also been previously reported, including expansion of the metalloprotease M35 family in *Coccidioides* (Li *et al.* 2012; Sharpton *et al.* 2009), expansion of genes encoding proteins with a peptidoglycan binding, lysin motif (LysM) domain in the dermatophytes (Martinez *et al.* 2012), and expansions of the fungal-specific protein kinase (FunK1) family independently in *Paracoccidioides* and *Coccidoides* (Desjardins *et al.* 2011).

To date, although representatives of all of the described pathogenic Onygenales genera have been sequenced, the only nonpathogenic species sequenced from this clade is *U. reesii*, which is estimated to have diverged from its closest known relatives, the two *Coccidioides* sp., 80 million years ago (Sharpton *et al.* 2009). This sequencing bias in favor of medically-relevant species is not uncommon, but phylogenomic studies that include related species with different lifestyles often reveal a very different picture of gene family changes and gene gain/loss than assumed with a more narrow selection of genomes of specific interest (Galperin and Koonin 2010). Here, we have sequenced four additional Onygenales species not known to be pathogenic, all of which are more closely related to *Coccidioides* spp. than to *U. reesii*. These additional genomes have allowed us to critically examine the extent of gene gain and loss, and gene family expansion and contraction in fungi.

We have focused our analyses on insights into two attributes of *Coccidioides* spp.: its ability to cause disease and its unique growth form in mammalian hosts. These additional genomes have also provided insights into the other Onygenales, particularly in gene family expansion/contraction analyses, showing that dense taxon sampling enhances phylogenomics just as it has benefitted phylogenetics. Adding additional genomes closely related to *Coccidioides* increased our confidence in finding genes unique to *Coccidioides*. Comparison with previous research shows that these unique genes have significantly fewer known protein domains and display increased expression in the unique endosporulating spherule of the parasitic phase (Whiston *et al.* 2012). More generally, our results indicate that individual gene gain/loss can play a significant role in branch-specific phenotypic change, such as the development of endosporulating spherules in *Coccidioides*. If the gene gain/loss trend continues for a period long enough to cause gene family expansion/contraction, phylogenetic radiations can occur that spawn entire orders with new, adaptive capabilities—such as the Onygenales, with their ability to exist on animal protein.

## Materials and Methods

### Strains and growth conditions

The following isolates were obtained from the University of Alberta Mycological Herbarium: *Byssoonygena ceratinophila* (UAMH 5669), *Amauroascus mutatus* (UAMH 3576), and *Amauroascus niger* (UAMH 3544). *Chrysosporium queenslandicum* (CBS 280.77) was obtained from the CBS-KNAW Fungal Biodiversity Centre. For genomic DNA isolation, isolates were grown on Sabouraud glucose agar (Kane *et al*. 1997) with cellophane (Bio-Rad Laboratories, Hercules, CA) for 14 days at room temperature, and snap frozen in liquid nitrogen. For total RNA isolation, isolates were grown on cellophane under a variety of conditions for 14 days, and snap frozen in liquid nitrogen: (i) Sabouraud glucose agar (Kane *et al*. 1997) at room temperature, (ii) Sabouraud glucose agar (Kane *et al*. 1997) at room temperature with a 1-hr incubation at 50°C prior to sample collection, (iii) Hay-infusion agar (CBS-KNAW 2012) at room temperature, and (iv) Oatmeal-salts agar (Kane *et al*. 1997) at room temperature.

### DNA extraction and library prep

To collect DNA, frozen mycelium was incubated in 6 ml lysis buffer (20 mM EDTA, 10 mM Tris-Cl, 1% Triton-X, 500 mM guanidine-Cl, 200 mM NaCl, 0.067 mg/ml chitinase, pH 7.9) for 1 hr at 37°C, then 4 µl RNase A (Qiagen, Valencia, CA), and 10 µl buffer G2 (Qiagen genomic quick tip kit) were added, followed by an additional 30-min incubation at 37°C. After the second incubation at 37**°**C, 30 µl proteinase K was added, followed by an overnight (15 hr) incubation at 50°C. Lysed mycelium was centrifuged at 4500 × *g* for 20 min to pellet cellular debris, and the supernatant was collected. To clean the DNA, three rounds of chloroform extractions, and one phenol-chloroform extraction were performed, each followed by an isopropanol precipitation with 70% ethanol washes. The final pellet was suspended in 80 µl TE buffer.

Illumina paired-end libraries were prepared using the standard Illumina Paired-End DNA Sample Prep Kit (Illumina Inc., San Diego, CA) protocol with a starting amount of 5 µg purified DNA. DNA was fragmented using the kit nebulizer for 6 min. Size selection (700 bp band) was performed postadapter ligation using a 2% low-melting-point agarose gel (Cambrex Bio Science, Rockland, ME). Following size selection, PCR (10 rounds) was performed with a starting amount of 0.25–0.35 ng DNA. Library size (average fragment size 700–750 bp) was confirmed by bioanalyzer at the Functional Genomics Laboratory, U.C. Berkeley.

### RNA extraction and library prep

Total RNA was extracted and purified using a previously described protocol (Kasuga *et al.* 2005). In brief, mycelium grown on cellophane was homogenized with 0.5 mm silica beads (Biospec, Bartlesville, OK) and Trizol (Invitrogen, Grand Island, NY) using a Mini Bead Beater (Biospec). Samples were then shaken at room temperature for nucleosome removal and further homogenized with chloroform on a Mini Bead Beater. Following centrifugation, the supernatant was collected, and total RNA was precipitated from the sample using isopropanol. RNA was suspended in nuclease-free water and 2 U/µl RNase OUT (Invitrogen) was added. RNA was further purified using the RNeasy mini kit (Qiagen). Total RNA integrity was confirmed by bioanalyzer at the Functional Genomics Laboratory, U.C. Berkeley.

Illumina paired-end libraries were prepared using the standard TruSeq PE RNA Sample Prep Kit (Illumina Inc.) protocol using a starting amount of 2–4 µg total RNA. Four conditions per isolate (see above) were prepared, indexed and combined into one sequencing lane per isolate; Illumina indexes 4 (i samples), 15 (ii samples), 6 (iii samples), and 7 (iv samples). Isolated mRNA was fragmented for 4 min at 94°C. Libraries were amplified using 10 rounds of PCR with a starting amount of 2–3 ng adapter-ligated cDNA fragments. Library size (average fragment size 300–400 bp) was confirmed by bioanalyzer at the Functional Genomics Laboratory, U.C. Berkeley; 100 bp, paired-end reads were sequenced on an Illumina HiSeq machine at the Vincent Coates Genome Sequencing Lab, U.C. Berkeley.

### Genome assembly and gene prediction

Prior to genome assembly, DNA reads were trimmed to the length where the mean quality score for the lane at that position was greater than 25. Genomes were assembled using the SOAPdenovo pipeline (Li *et al.* 2010): corrector, SOAPdenovo, GapCloser. Genes were predicted with the program MAKER (Cantarel *et al.* 2008) using the following parameters: gene models generated from RNA reads using Trinity (Grabherr *et al.* 2011; Haas *et al.* 2013), *Co. immitis* isolate RS gene transcripts as outside reference, and gene predictions generated by SNAP (Johnson *et al.* 2008) (*Co. immitis*-trained) and Augustus (Stanke and Morgenstern 2005) (*Co. immitis*-trained). RNA reads were mapped to the genome using Tophat (Trapnell *et al.* 2009), and expression levels for predicted genes were determined using Cufflinks (Trapnell *et al.* 2010). Genes that did not show an FPKM level above 5.0 in any of the growth conditions were removed from downstream analyses.

### Ortholog analysis and tree building

Comparative analyses were conducted using these assemblies/gene models and the following previously released genome sequences and gene models: *Co. immitis* isolate RS (Sharpton *et al.* 2009), *Co. posadasii* isolate Silveira (Neafsey *et al.* 2010), *U. reesii* (Sharpton *et al.* 2009), *Microsporum gypseum* (Martinez *et al.* 20012, *Trichophyton rubrum* (Martinez *et al.* 2012), *Histoplasma capsulatum* isolate nam1 (Broad Institute of Harvard and MIT), *Paracoccidioides lutzii* (formerly *Paracoccidioides brasiliensis*) (Desjardins *et al.* 2011), *Neurospora crassa* isolate OR74A (Galagan *et al.* 2003), *Aspergillus fumigatus* isolate Af293 (Galagan *et al.* 2005), and *As. nidulans* FGSC A4 (Galagan *et al.* 2005). Ortholog groups were determined using OrthoMCL (Li *et al.* 2003).

**Figure 1:**
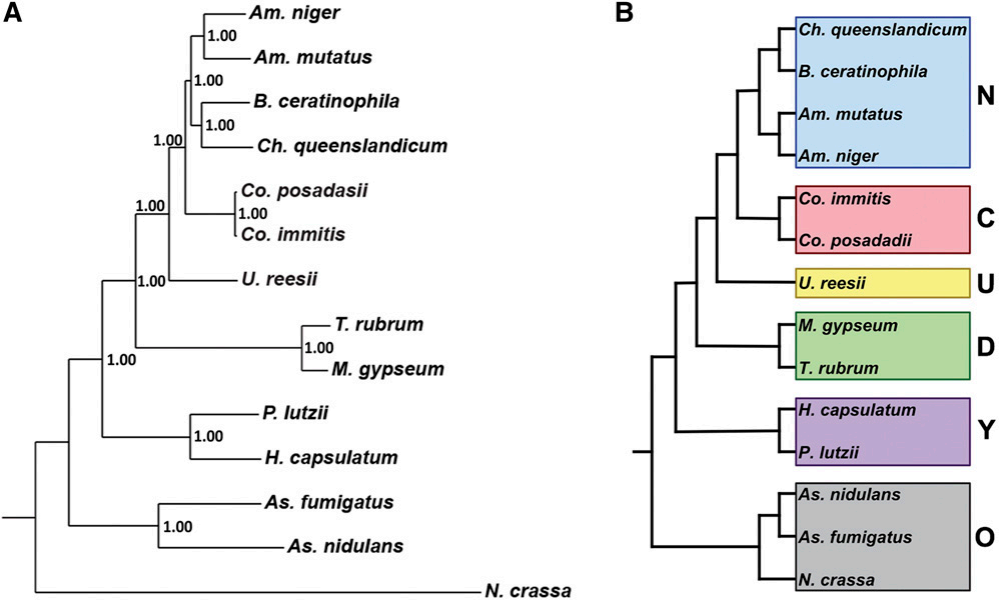
Phylogenetic distance tree (Bayesian) with posterior probabilities based on 100 randomly-selected single-copy orthologs (A). Phylogenetic categories used for gene family expansion/contraction and ortholog group analysis (B); N, newly sequenced genomes; C, *Coccidioides*, U*. reesii*; D, dermatophytes; Y, yeast-forming dimorphic fungal pathogens (YDFP); O, outgroups.

A species phylogenetic tree was generated using 100 randomly selected single-copy orthologs with representative sequences in all species. Individual genes sequences were aligned using MUSCLE (Edgar 2004) and the alignments were trimmed using trimAl (Capella-Gutierrez *et al.* 2009). The sequence alignments were concatenated and a Bayesian tree was generated using MrBayes (Ronquist and Huelsenbeck 2003) (GTR substitution model; gamma-distributed rate variation; 20,000 generations). This process was repeated with a set of single-copy housekeeping genes, and an additional 100 randomly selected single-copy orthologs to confirm the results. In addition, the species tree was further confirmed for the 100 randomly-selected ortholog gene alignment by a maximum likelihood tree generated using RAxML (Stamatakis 2006) executed in CIPRES (Miller *et al.* 2010) using the DNAGTRGAMMA model with 1000 bootstraps. All species trees generated provided the same topography reported in Figure 1.

### Pfam domain prediction and gene family analysis

Gene family analyses were conducted using the assemblies/gene models described above (novel and previously released). For consistency purposes in gene family comparison analyses, Pfam functional domains (Finn *et al.* 2010) were predicted for all genomes using hmmscan (HMMER 3.0, E-val < 0.001) (Eddy 2009). Gene family expansion/contraction analysis was carried out using CAFÉ (De Bie *et al.* 2006). Functional enrichment of gene groups of interest was carried out using GeneMerge (Castillo-Davis and Hartl 2003), and significance values were corrected using the Benjamini-Hochberg false discovery method (Benjamini and Hochberg 1995).

All genes with a predicted LysM domain from all genomes under comparison were aligned using MUSCLE (Edgar 2004). A gene tree was generated in RAxML (Stamatakis 2006), executed in CIPRES (Miller *et al.* 2010), using the PROTGAMMAWAG model with empirical frequencies and 1,000 bootstrap replicates; this was the best model for the gene alignment, as determined by ProtTest (Abascal *et al.* 2005).

### Selection analysis

Single copy ortholog groups were assessed for evidence of positive selection in *Am. mutatus*, *Am. niger*, *B. ceratinophila*, *Co. immitis*, *Co. posadasii*, *Ch. queenslandicum*, and *U. reesii*. Gene transcripts were aligned in coding triplets using TranslatorX (Abascal *et al.* 2010). Aligned genes were assessed for positive selection using the Branch Site REL model, implemented in HYPHY (Kosakovsky Pond *et al.* 2011).

### Data availability

All raw reads and assemblies are available in NCBI under BioProject PRJNA284880, BioSamples: SAMN03741935 (*Am. mutatus*), SAMN03741936 (*Am. niger*), SAMN03741937 (*B. ceratinophila*), and SAMN03741938 (*Ch. queenslandicum*). Raw reads are available under the following accession numbers for gDNA: SRS949549, SRS949625, SRS94954, and SRS949544; and for cDNA: SRX1044250, SRX1044252, SRX1044253, and SRX1044254. Genome assemblies (Whole Genome Shotgun) are available under the following master records: LJPJ00000000, LJPK00000000, LJPH00000000, and LJPI00000000. Transcript assemblies/gene models (Computationally Assembled Sequences) are available under the following accession numbers: GDQZ00000000, GDRA00000000, GDRB00000000, and GDRC00000000.

## Results

### Genome assembly and annotation

Illumina paired-end 101 bp reads were generated and assembled into draft genomes for *Amauroascus* (*Am*.) *mutatus*, *Am. niger*, *B. ceratinophila* and *Ch. queenslandicum*. Genome assemblies ranged in size from 29.9 to 37.7 Mb with N50s of 89–203 kb (Table 1). Predicted repetitive content was extremely low (0.4–1.0% of genome, Table 1), which is almost certainly an underestimate because repetitive regions do not assemble correctly using short read data (Paszkiewicz and Studholme 2010). Even without the expected amount of repetitive DNA, the newly sequenced genomes are slightly larger than that of *Co. immitis* RS, a finished genome, which is 28.9 Mb with 17% repetitive content.

**Table 1:** Genome assembly and annotation statistics. *N50: weighted median whereby 50% of the assembly is contained in contigs/scaffolds of this size or greater

		***Am. mutatus***	***Am. niger***	***B. ceratinophila***	***Ch. queenslandicum***	**Mean**
Genome (all contigs)	Total size (Mb)	31.6	37.7	29.9	33	**33**
	N50	203	94	89	172	**140**
	Total contigs/scaffolds	10,245	10,167	20,486	6480	**11,845**
	%GC	50	50	49	53	**50**
	Repetitive content (kb)/%	172 (0.55)	390 (0.99)	161 (0.57)	129 (0.39)	**213 (0.63)**
Genome (scaff > 0.5kb)	Total size (Mb)	30.4	36.7	27.4	32.3	**32**
	N50	218	99	103	174	**149**
	Total contigs/scaffolds	3078	3485	4867	2729	**3,540**
Genes	Total predicted	13,460	14,602	11,098	15,854	**13,754**
	Expressed (> 5 FPKM)	10,738	9132	9938	10,936	**10,186**
	% with Pfam domains (expressed)	71.5	70.8	71.3	69.6	**70.8**

For annotation, genes were predicted bioinformatically and confirmed by comparison to RNAseq data. The number of genes predicted per species by *ab initio*, homology-based and RNAseq assembly methods ranged from 11,098 to 15,854. From this total number of predicted genes, a mean of 10,186 genes showed evidence of expression (Table 1); of these, a mean of 71% had at least one recognizable Pfam domain.

### Phylogenetic tree

Ortholog groups were generated for *Am. mutatus*, *Am. niger*, *B. ceratinophila*, *Ch. queenslandicum*, and the following previously released genomes: *Co. immitis* RS, *Co. posadasii* Silveira, *U. reesii*, *M. gypseum*, *T. rubrum*, *H. capsulatum* nam1, *P. lutzii*, *As. fumigatus*, *As. nidulans*, and *N. crassa*. Of 1195 total all-species single-copy ortholog groups, 100 were randomly chosen to generate a phylogenetic tree (Figure 1A). The newly sequenced genomes, *Am. mutatus*, *Am. niger*, *B. ceratinophila*, and *Ch. queenslandicum* are all more closely related to *Coccidioides* spp. than to *U. reesii*. For the analyses described below, species were grouped into categories based on the phylogenetic tree: new genomes (*Am. mutatus*, *Am. niger*, *Ch. queenslandicum*, and *B. ceratinophila*), Coccidioides (*Co. immitis* and *Co. posadasii*), Uncinocarpus (*U. reesii*), dermatophytes (*M. gypseum* and *T. rubrum*), yeast-forming dimorphic fungal pathogens (YDFP; *H. capsulatum* and *P. lutzii*) and out groups (*N. crassa*, *As. nidulans* and *As. fumigatus*) (Figure 1B).

### Gene family expansion and contraction

Across all genomes, 325 Pfam domain categories showed evidence of changes in gene family size (Supporting Information, Table S1); gene families of interest are shown in Table 2. Gene family expansion and contraction has been previously assessed in the Onygenales (Desjardins *et al.* 2011; Sharpton *et al.* 2009), and it has been shown in the entire clade that gene families related to plant cell wall deconstruction have contracted, while gene families related to digesting animal material have expanded. This observation holds true for the genomes sequenced here, where we found contractions in the cellulose, fungal cellulase and glycosyl hydrolase families, and expansions in the subtilase and choline/ethanolamine kinase families, among others (Table 2).

**Table 2:** Significant (*P* ≤ 0.05) gene family expansion/contractions of interest

**Pfam Domain**	**Nc**	**Asf**	**Asn**	**Pl**	**Hc**	**Tr**	**Mg**	**Ur**	**Cop**	**Coi**	**Amn**	**Amm**	**Bc**	**Chq**
Cellulase (glycosyl hydrolase family 5)	5	**11**	**14**	2	3	2	1	2	1	1	5	5	4	1
Fungal cellulose binding domain	**21**	**17**	**6**	0	0	0	0	0	0	0	0	0	0	0
Glycosyl hydrolase family 61	**14**	**7**	**10**	0	0	0	0	0	0	0	0	0	0	0
Heterokaryon incompatibility protein (HET)	**63**	**7**	**7**	**6**	4	3	4	1	1	1	3	3	2	1
Taurine catabolism dioxygenase TauD	**13**	**14**	**21**	3	2	4	4	5	5	5	5	6	6	6
LysM domain	**8**	**8**	**18**	2	3	**13**	**22**	**8**	4	3	**13**	**14**	5	**8**
Choline/ethanolamine kinase	2	4	2	3	4	**15**	**12**	**9**	**9**	**8**	**28**	**91**	**58**	**74**
Deuterolysin metalloprotease (M35) family	3	3	4	1	1	**5**	**5**	**5**	**9**	**9**	3	**7**	4	**7**
Lipopolysaccharide kinase (Kdo/WaaP) family	6	6	4	5	**10**	**14**	**8**	**8**	**13**	**10**	**66**	**46**	**62**	**68**
Phosphotransferase enzyme family	33	47	36	40	**98**	**104**	**99**	**122**	**113**	**115**	**258**	**140**	**204**	**226**
Protein tyrosine kinase	80	81	82	78	79	85	82	**93**	**91**	**99**	**244**	**178**	**222**	**268**
Subtilase family	10	6	3	5	8	**18**	**16**	**19**	**19**	**18**	**22**	**31**	**25**	**30**

Expanded gene families indicated in bold; Columns by phylogenetic category: outgroups – *N. crassa* (Nc), *As. fumigatus* (Asf), *As. nidulans*; yeast-forming dimorphic fungal pathogens (YDFP) – *P. lutzii* (Pl), *H. capsulatum* (Hc); dermatophytes – *T. rubrum* (Tr), *M. gypseum* (Mg); *U. reesii* (Ur); *Coccidioides* – *Co. posadasii* (Cop), *Co. immitis* (Coi); new genomes – *Am. mutatus* (Amm), *Am. niger* (Amn), *B. ceratinophila* (Bc), *Ch. queenslandicum* (Chq)

**Figure 2:**
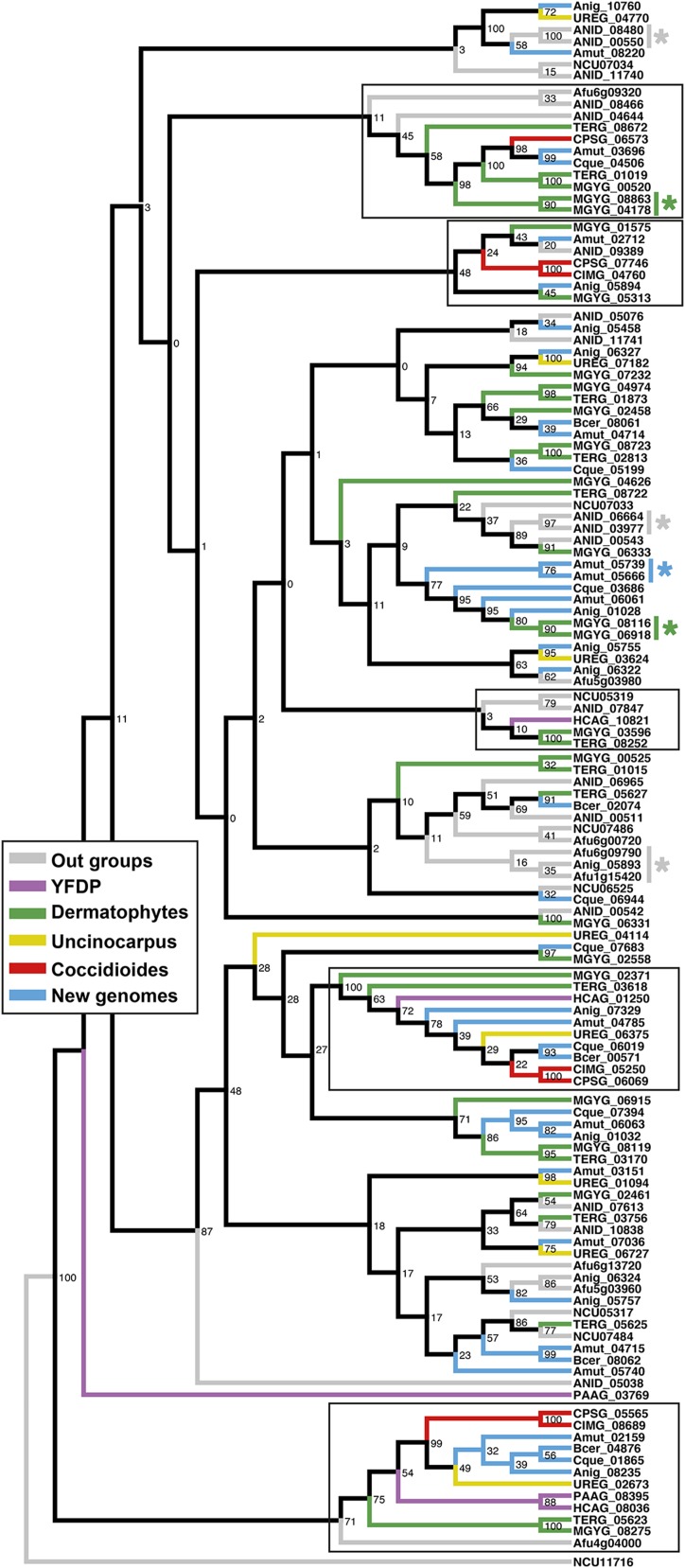
Gene family tree (maximum likelihood) with bootstrap values of all LysM domain-containing genes from all genomes used in this study. Branches with a LysM domain-containing gene in at least one of the Coccidioides or yeast-forming dimorphic pathogen (YDFP) species are outlined with a black box. Gene duplication events are indicated by the * symbol.

A previous comparative genomics study of onygenalean dermatophytes reported expansion of genes with at least one lysin motif (LysM) domain (Martinez *et al.* 2012). These proteins bind peptidogylcans, and have been shown to be involved in chitin sequestration in plant pathogenic fungi (de Jonge and Thomma 2009). Our results do not support LysM gene family expansion in the dermatophytes relative to the other Eurotiales. Instead, with additional genomes, we have observed a contraction of LysM genes in *Coccidioides* spp. and the two YDFP species (Table 2 and Figure 2). A phylogenetic tree for all genes containing at least one LysM domain shows only two gene duplications in the dermatophyte *M. gypseum* (not observed in *T. rubrum*); all other LysM genes in the dermatophytes have a homolog in at least one species from another phylogenetic branch (Figure 2). This tree also shows two well-defined branches where a LysM gene has been conserved in both *Coccdioides* and the YDFP, as well as LysM gene losses from both groups throughout the tree (Figure 2).

We observed additional gene family changes unrelated to the metabolism of plant and animal substrates (Table 2). There are fewer hetrokaryon incompatibility (HET) genes in all of the Onygenales, compared to *N. crassa* and *Aspergillus* spp., such that this family is restricted to just one locus in *U. reesii*, *Coccidioides* spp. and the new genomes. Our results also confirm a previous observation (Desjardins *et al.* 2011; Sharpton *et al.* 2009) that the deuterolysin metalloprotease (M35) family is expanded in *Coccioides* spp., but not in the genomes newly sequenced in this paper, or in the other Onygenales.

### Gene gain/loss and ortholog analysis

We assessed individual gene and loss patterns using ortholog group analysis in the Onygenales with reference to the *Coccidioides* spp. Based on gene gains and losses ascribed to the newly sequenced genomes, our gene prediction pipeline appears to neither overpredict, nor underpredict genes. Evidence that we have not underpredicted genes comes from ortholog group analysis (Table 3), where we observed the absence of just nine unique ortholog groups in the new genomes. Conversely, evidence that we have not overpredicted genes is provided by the relatively unremarkable number (420) of lineage-specific ortholog groups (genes shared by at least two taxa in a clade, and absent from other clades) found in the new genomes as compared to those found only in the dermatophyte clade (547 groups), or *Coccidioides* clade (791 groups).

**Table 3:** Summary of ortholog group analysis with differential expression (DE) data from Whiston *et al.* 2012

**Ortholog Groups**	**Include**	**Exclude**	**% w/Pfam Domain(s)**	***Co. immitis* DE**		
				**Up. Sap.**	**Up. Par.**	**No change**
5046	All	—	83.5	17.4	14.9	67.7
420	N	C, U, D, Y, O	56.0	—	—	—
9	C, U, D, Y, O	N	80.5	—	—	—
791	C	N, U, D, Y, O	7.4	3.4	29.1	67.5
41	N, U, D, Y, O	C	61.0	—	—	—
238	C, N	U, D, Y, O	32.4	10.1	19.4	70.5
10	U, D, Y, O	C, N	85.5	—	—	—
232	C, N, U	D, Y, O	33.3	12.9	20.7	66.4
90	D, Y, O	C, N, U	65.1	—	—	—
96	C, N, U, D	Y, O	57.8	16.3	13.3	70.4
222	Y, O	C, N, U, D	70.0	—	—	—
175	C, N, U, D, Y	O	45.4	16.3	20.5	63.2
1368	O	C, N, U, D, Y	64.6	—	—	—

Letter designations from Figure 1 used to designate phylogenetic groups: N, newly sequenced genomes; C, *Coccidioides*; U, *U. reesii*; D, dermatophytes; Y yeast-forming dimorphic fungal pathogens (YDFP); O, outgroups

We identified 791 lineage-specific ortholog groups in *Coccidioides*, that is, genes found in both *Co. immitis* and *Co. posadasii*, but no other species. A very small percentage, only 7.4%, of these lineage-specific genes had at least one predicted Pfam domain (Table 3), compared with a mean of 57.0% of all *Coccidioides* genes with at least one predicted domain. There were no Pfam domains significantly enriched in the *Coccioides* lineage-specific genes; this finding, and the very low percentage of unique genes with even one known Pfam domain, are to be expected from analysis of a set of unique genes.

As we expanded the taxonomic sampling by searching for ortholog groups that included *Coccidioides* and at least one representative from another phylogenetic category (newly sequenced nonpathogens, *U. reesii*, dermatophytes, and yeast-forming dimorphic fungal pathogens; see Figure 1), we found a higher mean percentage of genes with at least one predicted Pfam domain, which underscores the very small number of lineage-specific genes with a predicted domain (32–58% *vs.* 7.4% for *Coccidioides* alone, Table 3). Orthologs found only in *Coccidioides* and at least one of the newly sequenced nonpathogens were enriched for genes with an F-box domain, genes with the inclusion membrane domain IncA, and those in the phosphotransferase enzyme family. Orthologs found only in *Coccidioides*, the new genomes and *U. reesii* were enriched for six Pfam domains, including the subtilase family, and the deuterolysin metalloprotease (M35) family. Orthologs found only in *Coccidoides*, the new genomes, *U. reesii*, and the dermatophytes were enriched for genes in the phosphotransferase enzyme family. Ortholog groups found only in the Onygenales (*Coccidoides*, the new genomes, *U. reesii*, the dermatophytes, and the yeast-forming dimorphic fungal pathogens) were enriched for protein kinases, and the phosphotransferase enzyme family. Ortholog groups with at least one representative from each phylogenetic category had a high level of Pfam domain predictions—83.5% of genes had at least one predicted Pfam domain. There were 29 significantly enriched (*p*-value < 0.05) Pfam domains in this group of genes, among them many housekeeping functional domains, including helicase C-terminal domain, tyrosine kinase, DEAD box helicase, mitochondrial carrier protein, and ubiquitin carboxyl-terminal hydrolase domain. One would expect that gene family expansion influences estimates of functional enrichment revealed by ortholog comparison, an expectation confirmed by our analyses of choline kinases, phosphotransferases, subtilases and deuterolysin metalloproteases.

Genes identified as *Coccidioides* lineage-specific genes by ortholog analysis show a biologically interesting expression pattern. A recent study of gene expression in *Coccidioides* found that 9.0% of all genes in *Coccidioides* were significantly upregulated in the saprobic hyphal phase, and 13.5% of all genes were significantly upregulated in the parasitic spherule phase (Whiston *et al.* 2012). Of the 791 *Coccidioides* lineage-specific genes identified by ortholog analysis, only 3.4% were upregulated in the hyphal phase and 29.1% were upregulated in the spherule phase (Table 3). Given that the endosporulating spherule morphology is unique to *Coccidioides*, it makes biological sense that many genes critical to this growth form are found only in *Coccidioides*. The disparity between level of transcription in the two phases eroded as phylogenetic categories lacking spherules were added to the analysis, which resulted in an increasing percentage of genes that were upregulated in the saprobic phase (10–16%), and a decreasing percentage of genes that were upregulated in the parasitic phase (13–21%) (Table 3).

Nucleotide substitution rates were estimated for *U. reesii*, *Co. immitis*, *Co. posadasii*, *Am. mutatus*, *Am. niger*, *B. ceratinophila*, and *Ch. queenslandicum* using all-species single-copy ortholog groups. As expected based on the results of the phylogenetic tree, the sister species *Co. immitis* and *Co. posadasii* had the lowest median synonymous nucleotide substitution rate (dS, 0.22). Comparing *Co. immitis* to other species showed fully saturated dS (≥ 1.0). This saturation precludes dN/dS positive selection analysis, but not branch-site methods that compare likelihoods of evolutionary models with and without selection (*e.g.*, Yang and Nielsen 2002). However, the branch-site random effects likelihood (branch-site REL) tests for positive selection at individual sites on every branch of a given lineage, even when synonymous sites are saturated (Kosakovsky Pond *et al.* 2011). Using this test, we observed evidence of positive selection for 103 genes on the *Coccidioides* branch relative to other species (Table S2 and Figure S1). This gene set was enriched for five gene ontology (GO) terms: biological process (regulation of mRNA stability), cell wall (sensu Fungi), nickel ion binding, unfolded protein response and vacuole inheritance. Of the 103 genes showing evidence of positive selection in the *Coccidioides* branch, 19 genes were previously reported as upregulated in the parasitic spherule growth phase, and 21 were reported as upregulated in the saprobic hyphal growth phase (Whiston *et al.* 2012). These proportions of differential expression in genes showing evidence of positive selection are much higher than the differential expression rates for all genes in Coccidioides (13.5% up in spherule phase and 9% up in hyphal phase) (Whiston *et al.* 2012). The 40 genes showing evidence of positive selection and differential expression between growth phases would be interesting candidates for future hypothesis testing of allele swapping or gene deletion.

## Discussion

Gene family expansion/contraction and gene gain/loss have become recognized as important sources of genetic variation in fungi due to the advent of comparative phylogenomics. As with any type of comparative biology, the scope of phylogenetic sampling limits the outcome. Here, by sequencing the genomes of *B. ceratinophila*, *Ch. queenslandicum*, *Am. mutatus* and *Am. niger*, we increased the number of genomes of nonpathogenic Onygenales from one to five, and compared them to the genomes of six mammalian pathogens and three outgroup species. Our results support previous reports of the contraction of gene families involved in digesting plant cell walls throughout the Onygenales, and of the expansion of gene families involved in digesting animal protein in the *Coccidioides*–*Uncinocarpus* clade (Desjardins *et al.* 2011; Sharpton *et al.* 2009), but they contradict previous reports of the expansion in the dermatophytes of a gene family containing domains involved in binding peptidoglycans (Martinez *et al.* 2012).

### Gene family expansion/contraction

The peptidoglycan binding domain, LysM, is found throughout prokaryotes and eukaryotes (Buist *et al.* 2008). A previous study on comparative genomics, which focused on the dermatophytes, reported an expansion of proteins with the LysM domain in the dermatophytes *Microsporum* and *Trichopyton*, and speculated that these genes were involved in fungal immune evasion in the host environment (Martinez *et al.* 2012). Our analysis did not find frequent, conserved gene duplications in the dermatophytes as would be expected in a typical gene family expansion. Certainly, analysis of genes with LysM domains is complicated by the frequency of their apparent gain or loss in the dermatophytes (Martinez *et al.* 2012). However, with the exception of two gene duplication events in *M. gypseum*, all dermatophyte LysM genes have a homolog in at least one of the four newly sequenced genomes, and most dermatophyte LysM genes have a homolog in the outgroup, *Aspergillus*, as well. Gene duplication events are observed in *Am. mutatus* (one event, new genome), and *Aspergillus* spp. (three events, outgroup). Rather than a gene family expansion in dermatophytes, our results support separate, convergent gene family contractions in *Coccidioides*, and the yeast-forming dimorphic fungal pathogens. These results highlight the importance of sequencing as many genomes within a clade as possible for phylogenomics studies. Although *B. ceratinophila*, *Ch. queenslandicum*, *Am. mutatus* and *Am. niger* are not more closely related to the dermatophytes or yeast-forming dimorphic fungal pathogens than previously sequenced species from the Onygenales, these genomes have provided additional insights into the these groups.

LysM genes have a diverse and complex array of roles in fungi. It has been reported that these genes are involved in chitin sequestration in plant pathogens to aid evasion of the host immune response (de Jonge and Thomma 2009), and it was speculated that this gene family might play a similar role in the mammalian dermatophyte pathogens (Martinez *et al.* 2012). Given the convergent LysM gene family contractions in the internal pathogen groups relative to the nonpathogens and dermatophytic pathogens in the Eurotiales, this gene family may, in fact, be disadvantageous in internal, systemic infections. Rather than sequestering fungal cell wall polysaccharides from the immune system, perhaps the LysM proteins were, themselves, immunoreactive. Given that LysM domains are involved in binding to glycoprotein bonds, particularly to chitin monomers, the contractions observed in the dimorphic fungal pathogens might point to reduced fungal–fungal interactions throughout their life cycle. Furthermore, peptidoglycans are constituents of bacterial cell walls, so there could also be reduced fungal–bacterial interactions in the dimorphic fungal pathogens.

We also observed that the contraction of the HET incompatibility genes in the Onygenales, previously seen in *U. reesii* and the *Coccidioides* spp., extends to the newly sequenced genomes. HET genes are responsible for self-nonself recognition during nonsexual encounters in fungi. In Ascomycota, individuals can fuse to form heterokaryons only if they possess identical alleles at all *het* loci, thereby promoting self fusions and preventing nonself fusions. Heterokaryon incompatibility has been best studied in *Neurospora*, which has a very complex web of 63 genes with a HET domain (Table 2), and abundant evidence of frequency dependent selection that maintains trans-species polymorphism (Hall *et al.* 2010). Although *Coccidioides* spp. have only one HET gene, population genomics showed that its alleles have the high variability expected of genes under frequency-dependent selection, with the HET locus in *Co. immitis* showing 37 nonsynonymous SNPs) and that in *Co. posadasii* showing 15 nonsynonymous SNPs (Neafsey *et al.* 2010). Without tests of self-nonself interactions in *Coccidioides* species, it is not clear if these HET loci regulate hyphal fusion. If they do, as suggested by the allelic variation, then why is there only one locus, which could not reduce self-fusion to the low level enjoyed by Neurospora? Perhaps *U. reesii*, *Coccidioides* and the newly sequenced Onygenales genomes rarely encounter other members of their species in the environment, such that one, multi allelic locus suffices to regulate self-nonself recognition. It is worth noting, however, that the *Coccidioides* population is outbred (Burt *et al.* 1996; Fisher *et al.* 2000), and that core meiotic genes are expressed in *Coccidioides* spp. (Nguyen *et al.* 2013). It is also possible that the reduction of HET genes in *Coccidioides* and its relatives could relate to parasexuality; for example, parasexuality has been observed in the laboratory for *Aspergillus nidulans* (Pontecorvo 1956; Stromnaes and Garber 1963). However, *A. nidulans* also has a sexual cycle, and seven observed HET genes, and where sex has not been observed in fungi, particularly in BSL3 level pathogens, it has been due to a lack of observation rather than a lack of sex (O’Gorman *et al.* 2009; Taylor *et al.* 2015).

### Gene gain and loss

In addition to gene family changes, individual gene gain and loss has long been implicated in adaptation and evolution (Galperin and Koonin 2010). The number of genes, 791, gained in *Coccidioides* species is high in our analysis, despite our having added genomes of four, nonpathogenic fungi most closely related to *Coccidioides* spp., an addition that ought to reduce the number of genes unique to *Coccidioides* species. These 791 unique genes could be explained by the two obvious differences between *Coccidioides* species and their close relatives: parasitism, and the unique endosporulating spherule. Genetic control of parasitism or the unique growth form could be attributed to novel functions of genes studied in other species, genes not found or not yet studied in other species, or a combination of both. Comparing the results of this study with those from a recent transcriptomics study in *Coccidioides* (Whiston *et al.* 2012), a distinct trend emerges: genes unique to *Coccidioides* are strongly enriched for upregulation in the parasitic spherule growth phase, and have few functional domain predictions. This upregulation indicates that control of pathogenicity or spherule growth, or both, is largely controlled by genes of unknown function that are unique to *Coccdioides*, as opposed to novel function exhibited by genes of known function that have been studied in other fungi. Conversely, lineage-specific genes in *Coccidioides* do not appear to be critical to hyphal growth; this result is to be expected, because hyphal growth is the standard fungal growth form, and is shared by all the analyzed genomes. Hand-in-hand with these observations, lineage-specific genes in *Coccidioides* overwhelmingly (93%) encode proteins with no recognized domains or known functions. As the ortholog groups were systematically enlarged to include representative genes from more taxa, the percentage of recognized domains increased to a maximum of 84% when all the taxa were included.

### Positive selection

Recently, a branch-site model of positive selection was extended using a random effects likelihood (REL) framework to be able to identify evidence of site-based selection on any branch of a tree – both internal (interspecies) and background (interlineage) branches (Kosakovsky Pond *et al.* 2011). We were particularly interested in looking at positive selection in the *Coccidioides* branch, that is, selection acting on both *Co. immitis* and *Co. posadasii* in relation to its nearest relatives. We observed evidence of positive selection acting on the *Coccididioides* branch in 103 genes. The GO terms enriched in the positive selection set could be equally important for adaptation in either growth form—in particular, cell wall genes are critical to all growth phases, and unfolded protein response evolution could be vital to environment-specific stress response in either the soil or the host.

Although we expected a high number of these genes to be upregulated in the parasitic spherule growth phase, this group of genes was not enriched for genes previously reported to be upregulated in either growth phase (Whiston *et al.* 2012). One gene found to be under positive selection by our methods was proline-rich antigen (PRA, CIMG_09696)—a known antigen that has been extensively studied in *Coccidioides* as a possible vaccine candidate (Herr *et al.* 2007). PRA was previously shown to be under positive selection in a targeted gene sequencing study (Johannesson *et al.* 2004), which validates our methods here. Another positively-selected gene of interest for the spherule growth phase is urease accessory protein UreG (CIMG_05165). The urease pathway in *Coccidioides* produces extracellular ammonia during infection, and has been shown to contribute significantly to pathogenicity and host tissue damage (Mirbod-Donovan *et al.* 2006; Wise *et al.* 2013).

### Future directions

The goal of comparative genomics in the context of human pathogenic fungi is the prevention and control of fungal disease. Phylogenomics aims to identify genes whose function or history of natural selection indicates a pivotal role in pathogenesis. Our phylogenomic study enriches the field for future effort, by identifying 791 genes unique to *Coccidioides* species whose functions are largely unknown. To prioritize which of these genes are worthy of further study, an expansion of the previous population genomic study of *Coccidioides* seems warranted. The pioneering *Coccidioides* study employed 14 genomes (Neafsey *et al.* 2010), but subsequent research with many more strains in the model filamentous fungus *Neurospora* was very revealing. Regarding selection, analyses of very closely related populations of *Neurospora* led to discovery of genomic regions whose extreme divergence between, and uniformity within, populations indicated a recent history of natural selection. The genes in these regions suggested the environmental parameters important to adaptation, and hypotheses that two of the genes were adaptive survived testing by gene deletion in a process termed reverse ecology (Ellison *et al.* 2011). Regarding gene function, genome-wide association practiced on one of the aforementioned *Neurospora* populations uncovered nine genes associated with a complex trait where previous mutation and screening had found only two (Palma-Guerrero *et al.* 2013). Among the human pathogenic fungi, *Coccidioides* species are perhaps the best placed for population genomics, as already demonstrated (Neafsey *et al.* 2010), due to their well-characterized species and populations (Fisher *et al.* 2002; Koufopanou *et al.* 1997; Taylor and Fisher 2003) and large isolate collections (Barker *et al.* 2012). With the recent delisting of *Co. immitis* and *Co. posadasii* as select agents, we hope that more researcher groups will take advantage of natural variation in the culture collection to identify genes important to pathogenesis in *Coccidioides* species and then test those hypotheses by gene deletion and allele swapping experiments.

## Supplementary Material

Supporting Information
