# Supplementary material for: Comparative Phylogenomics of Pathogenic and Nonpathogenic Species
Source: G3 (Bethesda). 2015 Nov 25;6(2):235–44. doi: 10.1534/g3.115.022806 (PMC4751544; doi:10.1534/g3.115.022806)
Supplement: Supporting Information [file supp_6_2_235__index.html]

Comparative Phylogenomics of Pathogenic and Nonpathogenic Species — Supporting Information 

# Comparative Phylogenomics of Pathogenic and Nonpathogenic Species

## Supporting Information for Whiston and Taylor, 2016

**Files in this Data Supplement:**

- Figure S1 - Representative episodic selection tree generated in HyPhy (KOSAKOVSKY POND *et al.* 2011) for the CIMG\_11422 ortholog group, which shows positive selection (indicated in red) at the *Coccidioides* branch. (.pdf, 65 KB)
- Table S1 - Summary of all Pfam domain categories showing evidence of changes in gene family size. (.pdf, 168 KB)
- Table S2 - Summary of genes showing evidence of positive selection by the Branch Site REL test (KOSAKOVSKY POND *et al.* 2011). (.pdf, 150 KB)
