# Supplementary material for: Comparative Phylogenomics of Pathogenic and Nonpathogenic Species
Source: G3 (Bethesda). 2015 Nov 25;6(2):235–44. doi: 10.1534/g3.115.022806 (PMC4751544; doi:10.1534/g3.115.022806)
Supplement: Supporting Information [file supp_g3.115.022806_FigureS1.pdf]

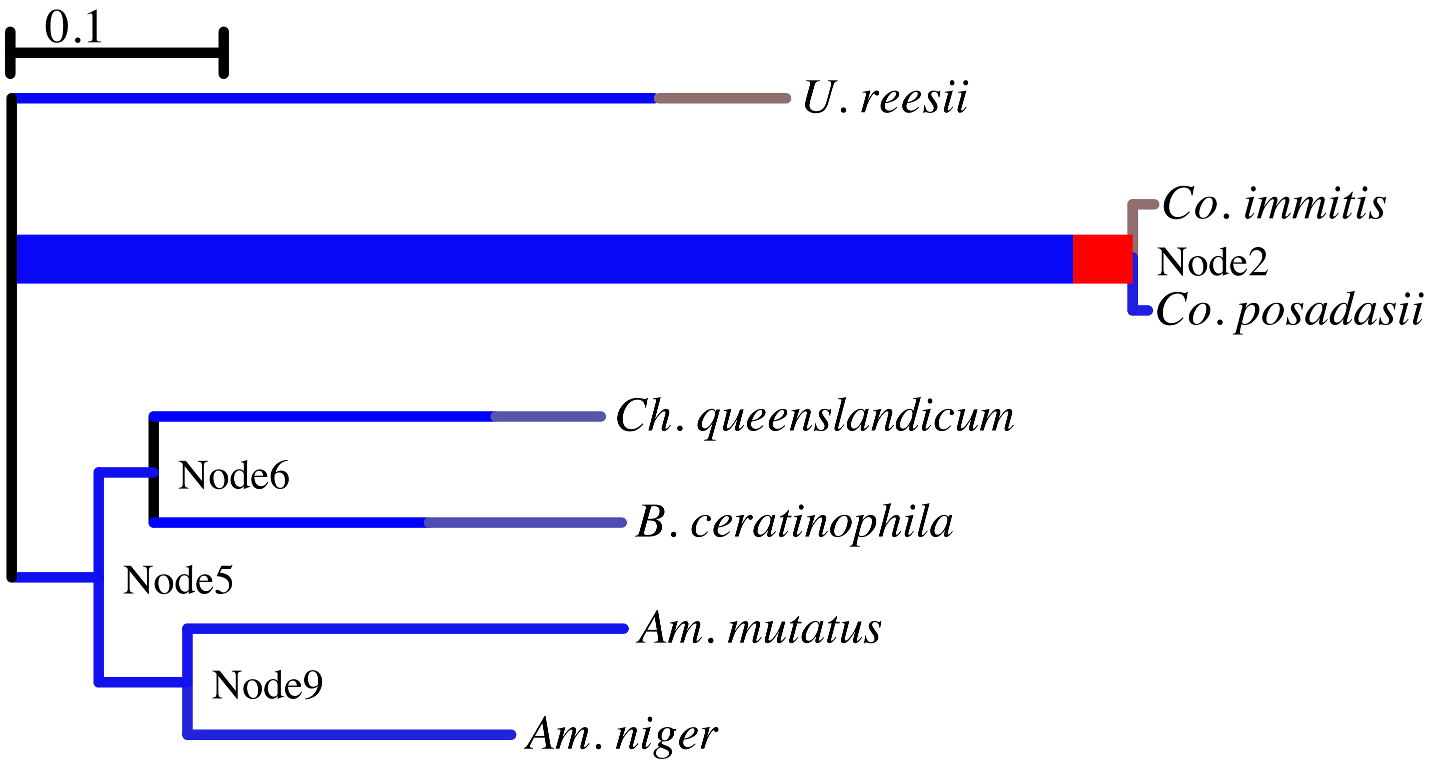

Figure S1. Representative episodic selection tree generated in HyPhy (KOSAKOVSKY POND *et al.* 2011) for the CIMG_11422 ortholog group, which shows positive selection (indicated in red) at the *Coccidioides* branch.
