# Supplementary material for: Comparative Phylogenomics of Pathogenic and Nonpathogenic Species
Source: G3 (Bethesda). 2015 Nov 25;6(2):235–44. doi: 10.1534/g3.115.022806 (PMC4751544; doi:10.1534/g3.115.022806)
Supplement: Supporting Information [file supp_g3.115.022806_TableS1.pdf]

Table S1. Summary of all Pfam domain categories showing evidence of changes in gene family size.

DESCRIPTION	ID	Ncra	Asfum	Asnid	Pbra	Hcap	Mgyp	Trub	Uree	Cimm	Cpos	Amut	Anig	Bcer	Cque	Tree-wide BH_Pval *
'chromo' (CHRromatin Organisation MOdifier)	Chromo	10	8	5	6	10	9	4	5	11	6	7	6	7	12	0.0184
2OG-Fe(II) oxygenase superfamily	2OG-FeII_Oxy	8	16	22	8	6	10	7	9	12	8	17	18	12	13	0.0184
3-beta hydroxysteroid dehydrogenase/isomeras	3Beta_HSD	7	17	13	3	5	6	7	3	6	7	31	29	25	29	0.0000
3-hydroxyacyl-CoA dehydrogenase, C-terminal	3HCDH	4	3	5	2	1	1	1	1	2	2	3	2	1	4	0.0307
3-hydroxyacyl-CoA dehydrogenase, NAD binding	3HCDH_N	7	10	12	3	3	4	5	7	5	6	14	19	13	24	0.0000
3-Oxoacyl-[acyl-carrier-protein (ACP)] synth	ACP_syn_III_C	0	0	1	1	0	1	1	1	1	1	3	6	0	7	0.0000
3-Oxoacyl-[acyl-carrier-protein (ACP)] synth	ACP_syn_III	2	1	4	1	1	2	1	3	4	3	6	8	4	9	0.0000
4-hydroxyphenylacetate 3-hydroxylase family	HpaB	0	0	0	0	0	0	0	0	0	0	1	2	1	4	0.0307
4Fe-4S binding domain	Fer4	3	2	2	2	2	2	2	1	2	2	4	3	1	6	0.0307
7 transmembrane receptor (rhodopsin family)	7tm_1	0	0	0	2	0	0	1	0	1	1	5	2	5	1	0.0000
A-macroglobulin complement component	A2M_comp	0	1	0	0	0	0	0	0	0	0	3	0	1	2	0.0184
ABC transporter	ABC_tran	48	61	65	39	40	56	49	44	49	47	89	69	78	82	0.0000
ABC-2 type transporter	ABC2_membrane	8	15	19	8	9	9	6	8	8	9	16	9	12	13	0.0000
Acetyltransferase (GNAT) family	Acetyltransf_1	31	45	44	25	30	38	35	38	41	39	54	39	45	48	0.0000
Acyl transferase domain	Acyl_transf_1	10	19	37	6	4	21	13	11	13	12	27	17	14	26	0.0000
Acyl-CoA dehydrogenase, C-terminal domain	Acyl-CoA_dh_1	9	10	17	12	11	10	10	12	13	12	22	25	19	26	0.0000
Acyl-CoA dehydrogenase, middle domain	Acyl-CoA_dh_M	9	12	17	16	8	10	10	15	15	14	22	26	21	28	0.0000
Acyl-CoA reductase (LuxC)	LuxC	0	1	0	0	0	1	1	1	0	0	3	5	2	7	0.0000
Adaptin N terminal region	Adaptin_N	8	8	8	8	10	8	8	8	8	8	25	17	18	23	0.0000
Adenovirus IVa2 protein	Adeno_IVa2	0	0	0	0	0	0	0	0	0	0	4	0	1	0	0.0000
Aflatoxin regulatory protein	AflR	0	1	3	0	0	0	0	0	1	1	3	0	0	3	0.0000
AhpC/TSA family	AhpC-TSA	9	12	12	7	7	8	6	4	6	5	10	13	11	18	0.0000
AIG2-like family	AIG2	1	3	5	1	0	1	1	4	3	3	2	5	7	4	0.0000
Alanine dehydrogenase/PNT, C-terminal domain	AlaDh_PNT_C	10	9	11	5	4	5	7	7	6	6	12	14	11	23	0.0000
Alcohol dehydrogenase GroES-like domain	ADH_N	29	53	68	23	21	29	24	27	23	24	47	32	37	43	0.0000
Aldehyde dehydrogenase family	Aldedh	14	23	28	13	12	14	13	14	14	14	24	24	18	23	0.0307
Aldo/keto reductase family	Aldo_ket_red	18	30	40	11	13	15	13	17	15	15	19	23	20	16	0.0000
alpha/beta hydrolase fold	Abhydrolase_1	42	51	49	28	26	37	36	28	33	32	39	36	37	38	0.0307
alpha/beta hydrolase fold	Abhydrolase_3	21	37	50	18	11	22	19	14	20	19	32	26	26	25	0.0000
Amidohydrolase family	Amidohydro_3	10	9	13	4	5	4	4	4	5	4	7	8	3	6	0.0450
Amidohydrolase family	Amidohydro_1	9	10	14	4	5	6	6	5	5	4	9	8	3	7	0.0000
Amino acid permease	AA_permease	17	40	51	18	18	30	32	28	29	28	48	39	34	42	0.0000
Aminotransferase class I and II	Aminotran_1_2	16	30	26	18	16	27	23	19	21	21	37	35	26	36	0.0000
AMP-binding enzyme	AMP-binding	27	51	68	38	42	51	46	43	39	42	62	59	50	56	0.0000
Archaeal ATPase	Arch_ATPase	1	2	3	1	0	2	2	2	2	2	45	31	30	32	0.0000
ArgK protein	ArgK	1	0	0	0	0	0	0	1	1	1	25	13	16	25	0.0000
Armadillo/beta-catenin-like repeat	Arm	16	19	21	15	15	15	15	10	16	17	21	13	21	17	0.0000
ATP-grasp domain	ATP-grasp_3	0	2	1	1	2	2	0	1	3	3	11	12	10	16	0.0450
ATP-grasp domain	ATP-grasp	6	5	6	4	4	3	4	5	5	5	9	14	9	16	0.0000
ATP-utilising chromatin assembly and remodel	WAC_Acf1_DNA_bd	2	1	1	1	2	1	1	0	1	1	2	1	0	3	0.0307
ATPase family associated with various cellul	AAA_5	46	47	42	45	65	53	45	38	44	41	77	64	68	64	0.0000
ATPase family associated with various cellul	AAA	59	52	53	48	74	52	48	48	51	49	97	83	86	80	0.0000
Bacterial alpha-L-rhamnosidase	Bac_rhamnosid	1	4	9	0	0	0	0	0	0	0	0	1	1	2	0.0000
Bacterial protein of unknown function (DUF894)	DUF894	0	0	1	0	0	0	0	0	0	0	1	5	5	0	0.0000
Bacterial protein of unknown function (DUF965)	DUF965	0	0	0	0	0	0	0	0	0	0	0	1	2	0	0.0307
Bacterial SH3 domain	SH3_3	0	2	0	0	0	1	0	0	0	0	2	1	0	2	0.0184
Bacterial transferase hexapeptide (three rep	Hexapep	9	10	11	8	7	7	9	6	7	8	11	5	9	9	0.0307
Bacteriophage lysis protein	Phage_lysis	0	0	0	0	0	0	0	0	0	0	0	1	2	0	0.0307
BCS1 N terminal	BCS1_N	3	3	3	1	20	2	3	2	1	2	5	5	1	1	0.0000
BED zinc finger	zf-BED	0	1	0	0	0	0	0	0	1	0	4	1	2	3	0.0307
Berberine and berberine like	BBE	12	14	24	3	5	11	12	3	4	4	5	10	4	9	0.0000
Beta-ketoacyl synthase, C-terminal domain	Ketoacyl-synt_C	9	17	38	5	2	22	13	9	12	13	30	20	13	24	0.0000
Beta-ketoacyl synthase, N-terminal domain	ketoacyl-synt	10	22	40	6	4	23	15	10	15	15	34	25	21	34	0.0000
Beta-lactamase	Beta-lactamase	6	6	8	4	3	8	6	7	5	6	12	4	5	6	0.0000
Beta-satellite pathogenicity beta C1 protein	Pathogen_betaC1	0	0	0	0	0	0	0	0	0	0	2	0	0	2	0.0184
BT1 family	BT1	0	2	0	0	0	0	0	0	0	0	2	1	3	0	0.0000
BTB/POZ domain	BTB	14	5	10	9	11	11	8	12	13	11	14	23	18	15	0.0000
bZIP transcription factor	bZIP_1	18	24	24	15	19	18	17	22	23	19	32	25	24	28	0.0307
Carbamoyl-phosphate synthase L chain, ATP bi	CPSase_L_D2	6	7	8	7	6	5	5	7	6	6	14	15	11	20	0.0184
Carbamoyl-phosphate synthase L chain, N-term	CPSase_L_chain	5	6	8	7	6	5	5	6	6	6	8	10	8	15	0.0184
Carboxylesterase	COesterase	12	20	27	7	4	12	12	4	6	6	13	12	8	10	0.0000

Carboxymuconolactone decarboxylase family	CMD	1	2	3	2	1	1	1	2	2	2	7	4	3	7	0.0184
Caspase domain	Peptidase_C14	2	2	3	1	1	3	2	1	1	1	1	5	8	5	0.0184
CDR ABC transporter	PDR_CDR	4	12	15	5	5	6	3	5	5	5	9	5	8	9	0.0184
Cellulase (glycosyl hydrolase family 5)	Cellulase	5	11	14	2	3	1	2	2	1	1	5	5	4	7	0.0184
CENP-B N-terminal DNA-binding domain	CENP-B_N	0	4	1	1	1	0	0	0	1	1	3	1	3	2	0.0307
Centromere protein B dimerisation domain	Cenp-B_dimeris	0	0	0	0	0	0	0	0	0	0	2	0	1	0	0.0307
Centromere protein H (CENP-H)	CENP-H	0	0	1	0	0	0	0	0	0	0	0	2	0	2	0.0000
CFEM domain	CFEM	13	3	7	6	3	7	6	7	10	8	14	8	12	15	0.0000
Chitin recognition protein	Chitin_bind_1	4	6	10	1	2	12	7	5	3	3	8	7	5	9	0.0000
Choline/ethanolamine kinase	Choline_kinase	2	4	2	3	4	12	15	9	8	9	28	91	58	74	0.0000
Chordopoxvirus fusion protein	Vac_Fusion	0	0	0	0	0	0	0	0	0	0	0	1	0	2	0.0307
Citrate synthase	Citrate_synt	4	5	3	2	2	2	2	2	2	2	9	3	4	3	0.0000
CoA-transferase family III	CoA_transf_3	4	5	6	4	2	2	2	5	6	6	7	8	5	12	0.0000
Cobalamin biosynthesis protein CobT	CobT	0	0	0	0	0	0	0	0	0	0	0	1	2	0	0.0307
Cobalamin synthesis protein cobW C-terminal	CobW_C	1	2	2	0	1	2	2	2	1	1	3	1	1	4	0.0307
CobW/HypB/UreG, nucleotide-binding domain	cobW	7	8	9	8	8	7	7	6	9	9	27	17	19	22	0.0000
Collagen triple helix repeat (20 copies)	Collagen	1	3	4	1	0	0	1	0	0	0	0	1	0	1	0.0307
Common central domain of tyrosinase	Tyrosinase	8	3	10	6	7	1	1	5	3	3	4	2	2	5	0.0000
Condensation domain	Condensation	3	15	19	4	6	18	15	13	7	9	18	19	12	16	0.0000
Copper amine oxidase, N2 domain	Cu_amine_oxidN2	1	6	5	0	2	1	2	5	6	6	7	5	5	7	0.0000
CorA-like Mg2+ transporter protein	CorA	11	6	11	8	6	6	6	4	5	5	11	8	7	10	0.0307
Core histone H2A/H2B/H3/H4	Histone	13	10	10	22	10	9	11	9	19	11	15	14	17	19	0.0000
Cupin	Cupin_1	2	4	9	1	0	3	3	1	2	3	3	4	5	6	0.0000
Cyclopropane-fatty-acyl-phospholipid synthas	CMAS	5	3	5	0	2	3	3	3	3	2	20	16	11	14	0.0184
Cys/Met metabolism PLP-dependent enzyme	Cys_Met_Meta_PP	7	10	6	6	6	7	7	8	7	8	16	22	14	21	0.0000
Cytochrome P450	p450	41	74	117	34	38	67	61	41	43	42	78	49	55	65	0.0000
D-ala D-ala ligase C-terminus	Dala_Dala_lig_C	1	3	1	1	4	5	2	2	3	2	12	13	11	19	0.0000
D-aminoacylase, C-terminal region	D-aminoacyl_C	0	0	0	0	0	0	0	0	0	0	1	0	2	0	0.0307
D-isomer specific 2-hydroxyacid dehydrogenas	2-Hacid_dh	9	10	16	8	8	8	8	7	11	9	14	11	9	13	0.0184
D-isomer specific 2-hydroxyacid dehydrogenas	2-Hacid_dh_C	11	14	20	8	9	10	9	8	12	10	22	26	22	28	0.0000
DDE superfamily endonuclease	DDE	0	48	3	13	0	0	1	0	2	5	1	2	0	1	0.0000
DEAD/DEAH box helicase	DEAD	52	51	48	42	53	50	47	46	48	48	94	76	96	95	0.0184
DegT/DnrJ/EryC1/StrS aminotransferase family	DegT_DnrJ_EryC1	3	4	1	2	1	3	3	2	2	2	15	16	10	16	0.0000
Dehydrogenase E1 component	E1_dh	3	5	3	3	4	3	3	4	4	4	5	7	6	11	0.0450
DeoR-like helix-turn-helix domain	HTH_DeoR	0	0	0	0	0	0	0	0	1	1	1	4	0	1	0.0184
Deuterolysin metalloprotease (M35) family	Peptidase_M35	3	3	4	1	1	5	5	5	9	9	7	3	4	7	0.0000
DivIVA protein	DivIVA	0	0	0	0	1	0	0	0	0	0	2	0	1	0	0.0184
DJ-1/PfpI family	DJ-1_PfpI	4	8	5	4	5	4	4	2	4	3	10	3	5	6	0.0000
DNA methylase	N6_N4_Mtase	0	0	0	0	0	0	0	0	0	0	1	0	3	0	0.0184
Domain of Unknown Function (DUF1087)	DUF1087	0	13	0	0	0	0	0	0	0	0	2	0	2	0	0.0000
Domain of unknown function (DUF2467)	DUF2467	10	17	24	9	7	6	6	6	5	6	13	11	14	12	0.0000
Elongation factor Tu GTP binding domain	GTP_EFTU	18	17	18	16	16	16	16	16	16	17	64	60	54	71	0.0000
Endonuclease/Exonuclease/phosphatase family	Exo_endo_phos	14	27	9	9	11	9	9	9	10	10	12	10	17	13	0.0000
Endoribonuclease L-PSP	Ribonuc_L-PSP	3	6	12	3	3	4	5	4	4	4	5	6	5	11	0.0000
Enoyl-CoA hydratase/isomerase family	ECH	7	12	15	14	13	10	9	11	14	12	17	21	14	23	0.0000
Equine arteritis virus serine endopeptidase	Peptidase_S32	0	0	0	0	0	0	0	1	0	0	2	0	0	5	0.0000
Ergosterol biosynthesis ERG4/ERG24 family	ERG4_ERG24	4	6	3	3	4	6	5	2	4	4	5	2	6	3	0.0184
Esterase PHB depolymerase	Esterase_phd	4	4	2	0	0	0	0	0	1	2	2	0	0	0	0.0307
Eukaryotic aspartyl protease	Asp	17	7	10	5	5	6	6	5	4	4	8	6	7	10	0.0307
Eukaryotic cytochrome b561	Cytochrom_B561	1	1	0	1	0	1	1	1	1	1	1	3	0	3	0.0000
FAD binding domain	FAD_binding_2	25	41	62	15	13	22	14	22	24	23	47	50	34	62	0.0000
FAD binding domain	FAD_binding_4	22	32	48	11	15	20	19	12	16	16	17	20	11	18	0.0000
FAD binding domain	FAD_binding_3	33	48	82	15	18	32	22	23	21	21	52	46	29	58	0.0000
FAD dependent oxidoreductase	DAO	55	86	116	35	33	47	41	44	48	46	85	83	67	93	0.0000
FAD-binding domain	FAD_binding_8	10	18	10	7	9	8	8	7	7	8	13	8	7	9	0.0000
Ferric reductase like transmembrane componen	Ferric_reduct	10	17	10	7	13	8	8	8	8	8	10	7	7	10	0.0000
Ferritin-like domain	Ferritin	0	0	0	0	0	0	0	0	0	0	0	1	0	2	0.0307
FG-GAP repeat	FG-GAP	0	2	5	0	0	0	0	0	0	0	3	0	0	0	0.0000
Flagellar basal body-associated protein FliL	FliL	3	4	0	0	1	0	4	0	1	2	0	1	0	0	0.0184
Flavin containing amine oxidoreductase	Amino_oxidase	18	28	35	7	10	14	11	16	17	17	23	23	15	25	0.0000
FR47-like protein	FR47	10	11	11	7	5	15	15	7	13	15	23	16	17	23	0.0000
Fringe-like	Fringe	0	0	1	0	0	0	0	0	0	0	3	2	1	4	0.0307
FtsJ-like methyltransferase	FtsJ	4	4	8	4	3	4	5	7	6	6	6	7	4	10	0.0000
Fungal cellulose binding domain	CBM_1	21	17	6	0	0	0	0	0	0	0	0	0	0	0	0.0000
Fungal fucose-specific lectin	Fungal_lectin	0	1	0	0	0	1	1	0	0	0	1	0	0	2	0.0184
Fungal specific transcription factor domain	Fungal_trans	65	134	177	43	48	65	57	51	55	54	91	65	69	80	0.0000
Fungal trichothecene efflux pump (TRI12)	TRI12	12	21	26	10	10	25	15	11	20	17	58	34	30	35	0.0000

Fungal Zn(2)-Cys(6) binuclear cluster domain	Zn_clus	99	186	246	52	55	104	92	60	95	83	133	107	101	115	0.0000
GAF domain	GAF	3	2	4	1	0	1	1	0	1	1	2	1	3	6	0.0000
Glucose inhibited division protein A	GIDA	7	10	11	3	2	6	6	6	4	5	26	30	19	37	0.0000
Glutamine amidotransferase class-I	GATase	10	8	9	8	7	8	9	9	10	8	15	15	12	23	0.0000
Glutaredoxin	Glutaredoxin	4	6	5	4	3	4	4	4	4	5	6	9	4	8	0.0307
Glutathione S-transferase, C-terminal domain	GST_C	10	17	17	6	7	11	14	10	8	8	15	16	13	19	0.0000
Glutathione S-transferase, N-terminal domain	GST_N	6	15	12	3	6	10	12	8	7	7	11	10	8	13	0.0000
Glyceraldehyde 3-phosphate dehydrogenase, NA	Gp_dh_N	1	3	2	1	2	1	1	1	1	1	2	4	1	4	0.0307
Glycophorin A	Glycophorin_A	0	0	0	0	0	0	0	0	0	0	2	0	0	1	0.0307
Glycosyl hydrolase family 3 C terminal domai	Glyco_hydro_3_C	8	16	19	3	5	4	4	3	3	3	8	6	9	7	0.0000
Glycosyl hydrolase family 3 N terminal domai	Glyco_hydro_3	10	18	21	4	4	6	7	5	5	5	9	6	8	7	0.0000
Glycosyl hydrolase family 61	Glyco_hydro_61	14	7	10	0	0	0	0	0	0	0	0	0	0	0	0.0000
Glycosyl Hydrolase Family 88	Glyco_hydro_88	2	4	6	2	1	0	0	0	2	2	1	0	1	0	0.0307
Glycosyl hydrolase family 92	Glyco_hydro_92	2	4	5	2	1	2	2	2	1	1	3	5	2	5	0.0184
Glycosyl hydrolases family 18	Glyco_hydro_18	11	17	20	5	9	17	13	13	8	7	19	19	7	12	0.0000
Glycosyl hydrolases family 2, immunoglobulin	Glyco_hydro_2	4	2	8	1	0	0	0	1	0	0	2	1	1	2	0.0000
Glycosyl hydrolases family 2, sugar binding	Glyco_hydro_2_N	3	5	9	0	1	2	2	2	0	0	2	1	1	2	0.0184
Glycosyl hydrolases family 2, TIM barrel dom	Glyco_hydro_2_C	2	1	7	0	0	1	1	0	0	0	3	3	3	3	0.0184
Glycosyl hydrolases family 28	Glyco_hydro_28	2	13	11	0	0	0	0	0	0	0	0	0	0	0	0.0000
Glycosyl hydrolases family 43	Glyco_hydro_43	5	18	17	1	2	1	1	1	1	1	1	1	0	0	0.0000
GMC oxidoreductase	GMC_oxred_N	10	13	27	9	9	5	4	6	6	6	6	10	9	7	0.0000
GMC oxidoreductase	GMC_oxred_C	8	14	24	7	9	5	4	3	5	5	5	8	6	5	0.0000
GPR1/FUN34/yaaH family	Grp1_Fun34_YaaH	1	1	6	2	2	2	2	2	2	3	4	4	1	4	0.0184
GRF zinc finger	zf-GRF	2	1	0	1	0	1	1	2	3	2	2	4	2	5	0.0307
GTPase of unknown function	MMR_HSR1	52	51	50	35	35	49	43	39	44	41	104	90	85	100	0.0000
HEAT repeat	HEAT	47	48	52	46	50	47	45	44	49	47	63	50	70	65	0.0307
Helicase conserved C-terminal domain	Helicase_C	75	80	72	68	72	77	72	67	73	73	100	84	103	104	0.0307
helix-turn-helix, Psq domain	HTH_psq	1	30	0	2	1	2	0	1	0	3	1	2	1	1	0.0000
Herpesvirus glycoprotein D	Herpes_glycop_D	7	4	5	5	4	4	3	2	3	3	2	3	1	7	0.0000
Heterokaryon incompatibility protein (HET)	HET	63	7	7	6	4	4	3	1	1	1	3	3	2	2	0.0000
HI0933-like protein	HI0933_like	13	24	32	6	3	11	8	9	12	11	27	36	23	44	0.0000
Homeobox associated leucine zipper	HALZ	0	2	1	1	4	3	3	0	3	3	5	3	1	3	0.0000
Hydantoinase/oxoprolinase N-terminal region	Hydant_A_N	2	1	6	2	3	3	3	3	3	3	7	4	5	5	0.0184
Hydrogenase expression/synthesis hypA family	HypA	0	1	0	0	1	0	0	0	1	1	4	1	0	1	0.0000
Importin-beta N-terminal domain	IBN_N	10	8	8	8	7	9	8	9	9	9	15	8	16	16	0.0307
Integrase core domain	rve	0	7	0	16	3	3	1	0	0	0	0	0	0	2	0.0000
Interferon-inducible GTPase (IIGP)	IIGP	2	1	1	1	2	3	2	2	1	1	4	1	4	1	0.0184
Intermediate filament protein	Filament	2	0	0	1	0	0	0	0	1	0	12	8	10	7	0.0184
Intermediate filament tail domain	IF_tail	0	0	0	0	0	0	0	0	0	0	0	0	0	4	0.0184
Ion channel	Ion_trans_2	2	3	0	1	0	1	1	1	0	0	0	1	1	0	0.0307
KR domain	KR	44	65	77	19	17	42	29	21	31	32	98	92	69	99	0.0000
L-fucokinase	Fucokinase	0	0	1	0	0	0	0	0	0	0	3	0	0	0	0.0450
Lipopolysaccharide core biosynthesis protein (	WaaY	0	0	0	0	0	0	0	0	0	0	2	7	4	3	0.0000
Lipopolysaccharide kinase (Kdo/WaaP) family	Kdo	6	6	4	5	10	8	14	8	10	13	46	66	62	68	0.0000
Lycopene cyclase protein	Lycopene_cycl	15	23	39	7	15	14	9	14	12	10	32	32	16	33	0.0000
LysM domain	LysM	8	8	18	2	3	22	13	8	3	4	15	11	6	10	0.0000
MAC/Perforin domain	MACPF	0	0	1	0	0	1	1	1	0	0	4	1	3	1	0.0184
Major Facilitator Superfamily	MFS_1	167	325	406	118	134	187	168	152	164	156	226	167	166	177	0.0000
Malate:quinone oxidoreductase (Mqo)	Mqo	0	2	0	0	0	1	1	0	1	1	0	1	0	1	0.0307
Male sterility protein	NAD_binding_4	10	19	33	8	10	17	14	7	11	11	23	23	14	23	0.0000
MATH domain	MATH	1	0	2	1	0	1	1	0	1	1	2	4	1	2	0.0307
Matrixin	Peptidase_M10	0	0	1	0	0	2	1	2	1	1	1	3	1	6	0.0000
MbeD/MobD like	MbeD_MobD	0	0	0	0	2	0	0	0	0	1	2	0	1	0	0.0000
Melibiase	Melibiase	0	7	6	1	0	0	0	0	0	0	1	1	2	1	0.0000
Metallo-beta-lactamase superfamily	Lactamase_B	13	17	20	9	9	11	11	11	11	8	19	14	14	17	0.0184
Methionine biosynthesis protein MetW	MetW	2	4	1	1	2	4	3	5	2	2	6	4	3	8	0.0000
Methyltransferase domain	Methyltransf_11	67	56	68	40	46	72	68	47	57	55	87	75	67	80	0.0000
Methyltransferase domain	Methyltransf_12	64	57	69	40	43	65	59	51	55	53	81	77	68	79	0.0184
Methyltransferase small domain	MTS	16	11	10	8	10	13	12	14	15	13	42	41	31	41	0.0307
Microtubule-binding protein MIP-T3	MIP-T3	1	0	0	0	0	2	0	0	0	0	2	2	1	5	0.0307
Miro-like protein	Miro	60	62	53	46	47	57	53	48	58	53	99	85	87	97	0.0184
Molybdopterin oxidoreductase	Molybdopterin	1	2	3	1	3	1	1	2	1	1	4	4	2	6	0.0307
Molybdopterin oxidoreductase Fe4S4 domain	Molybdop_Fe4S4	0	0	1	0	0	0	0	0	0	0	1	0	0	3	0.0000
Mucin-like glycoprotein	Mucin	0	2	0	0	2	1	0	0	0	0	3	1	2	1	0.0000
Mur ligase family, glutamate ligase domain	Mur_ligase_C	0	2	0	1	0	0	0	0	0	0	1	2	0	1	0.0307
Mur ligase middle domain	Mur_ligase_M	1	3	1	1	4	1	1	0	1	1	4	4	3	6	0.0184
Myb-like DNA-binding domain	Myb_DNA-binding	17	17	24	19	31	21	20	15	20	19	29	23	21	22	0.0000
MYND finger	zf-MYND	6	3	3	2	4	7	5	3	5	4	2	7	7	10	0.0000

N-Acetylglucosaminyltransferase-IV (GnT-IV)	Glyco_transf_54	0	0	2	0	0	0	3	1	0	0	2	1	0	2	0.0307
NACHT domain	NACHT	25	27	32	15	18	30	21	16	20	17	58	48	39	54	0.0000
NAD binding domain of 6-phosphogluconate deh	NAD_binding_2	7	9	10	3	5	7	7	5	6	7	22	18	12	19	0.0000
NAD dependent epimerase/dehydratase family	Epimerase	33	57	69	20	21	31	24	9	24	23	60	67	48	69	0.0000
NAD-dependent glycerol-3-phosphate dehydroge	NAD_Gly3P_dh_N	2	5	3	1	1	1	3	3	2	2	11	7	3	8	0.0000
NAD(P) transhydrogenase beta subunit	PNTB	1	1	1	1	2	1	1	1	1	1	2	5	0	3	0.0000
NADP oxidoreductase coenzyme F420-dependent	F420_oxidored	7	12	17	1	4	2	4	2	8	7	5	8	7	10	0.0000
NADPH-dependent FMN reductase	FMN_red	0	4	1	0	0	1	0	0	1	1	1	0	1	2	0.0184
NB-ARC domain	NB-ARC	9	4	8	4	10	11	6	6	7	5	29	23	17	29	0.0000
NmrA-like family	NmrA	15	29	39	8	10	10	11	11	13	14	29	30	23	27	0.0000
NNMT/PNMT/TEMT family	NNMT_PNMT_TEMT	1	3	0	0	4	1	1	0	0	0	2	2	2	2	0.0000
Nuclear RNA-splicing-associated protein	SR-25	2	0	1	1	3	0	0	1	1	1	4	1	0	3	0.0000
Nucleoplasmin	Nucleoplasmin	0	0	0	1	0	0	0	0	0	0	1	0	4	1	0.0184
O-methyltransferase	Methyltransf_2	7	10	16	2	2	9	12	4	6	6	9	8	6	7	0.0184
Outer membrane protein (OmpH-like)	OmpH	1	1	0	2	1	0	0	1	0	0	2	0	0	4	0.0000
Oxidoreductase family, NAD-binding Rossmann	GFO_IDH_MocA	7	12	19	5	5	7	7	4	4	4	11	9	6	7	0.0184
PAN-like domain	PAN_2	0	1	0	0	0	0	0	0	0	0	0	0	4	0	0.0184
PAS fold	PAS	8	3	3	4	2	1	1	0	2	3	5	5	1	4	0.0307
PBS lyase HEAT-like repeat	HEAT_PBS	3	3	5	3	8	2	2	1	4	4	8	4	7	12	0.0000
PcrB family	PcrB	1	0	0	0	1	0	0	0	0	0	1	1	0	3	0.0450
Peptidase C26	Peptidase_C26	4	6	5	4	5	5	5	5	7	6	10	13	9	20	0.0000
Peptidase family S64	Peptidase_S64	0	0	0	0	0	0	2	1	0	0	2	0	1	5	0.0000
Permease for cytosine/purines, uracil, thiam	Transp_cyt_pur	3	5	12	5	2	7	6	4	4	4	7	3	4	4	0.0000
Phosphopantetheine attachment site	PP-binding	9	34	42	8	11	30	27	17	17	19	35	32	19	30	0.0000
Phosphorylase superfamily	PNP_UDP_1	4	10	24	5	6	2	2	1	3	3	4	3	4	2	0.0000
Phosphotransferase enzyme family	APH	33	47	36	40	98	99	104	122	115	113	140	258	204	226	0.0000
Phytanoyl-CoA dioxygenase (PhyH)	PhyH	4	10	19	3	3	7	4	5	5	5	4	3	4	4	0.0000
Plant protein of unknown function (DUF869)	DUF869	0	0	0	0	0	0	0	0	0	0	0	4	1	1	0.0184
Polysaccharide biosynthesis protein	Polysacc_synt_2	3	5	6	0	2	1	2	1	1	1	20	16	11	20	0.0000
Poxvirus virion envelope protein A14	Pox_A14	0	0	0	0	0	0	0	0	0	0	4	0	2	0	0.0000
PPR repeat	PPR	10	9	16	8	12	12	11	10	10	11	16	15	17	12	0.0000
Predicted membrane protein (DUF2207)	DUF2207	0	1	0	0	0	0	1	1	0	0	4	0	0	1	0.0000
Predicted membrane protein (DUF2306)	DUF2306	0	0	0	0	0	2	2	0	0	0	2	1	0	3	0.0184
Prenyltransferase and squalene oxidase repea	Prenyltrans	5	10	9	4	5	5	4	4	5	6	8	6	5	9	0.0450
PrkA AAA domain	AAA_PrkA	0	2	1	0	0	0	0	0	0	0	3	1	3	1	0.0184
Prolyl oligopeptidase family	Peptidase_S9	10	15	21	5	6	7	8	9	10	9	25	22	29	24	0.0000
Protein kinase domain	Pkinase	107	114	104	111	152	139	144	165	153	146	214	299	260	306	0.0000
Protein of unknown function (DUF1112)	DUF1112	0	0	0	0	0	0	0	0	0	0	2	0	0	1	0.0307
Protein of unknown function (DUF1155)	DUF1155	0	0	1	0	0	0	0	0	0	0	0	1	0	2	0.0184
Protein of unknown function (DUF1515)	DUF1515	0	0	0	0	0	0	0	0	0	0	1	0	0	2	0.0307
Protein of unknown function (DUF1664)	DUF1664	0	1	1	0	1	0	0	0	0	0	2	3	4	0	0.0000
Protein of unknown function (DUF1679)	DUF1679	0	0	0	0	1	2	3	2	0	1	2	13	12	7	0.0000
Protein of unknown function (DUF2029)	DUF2029	0	0	0	0	0	0	0	0	0	1	1	0	2	0	0.0307
Protein of unknown function (DUF342)	DUF342	0	0	2	0	1	1	0	0	0	0	0	1	0	1	0.0184
Protein of unknown function (DUF904)	DUF904	2	0	3	0	0	0	1	0	1	2	1	6	1	4	0.0000
Protein of unknown function (DUF972)	DUF972	2	1	1	0	1	1	0	0	1	1	0	3	1	2	0.0184
Protein of unknown function, DUF258	DUF258	20	21	29	14	16	21	15	12	20	16	77	65	62	71	0.0000
Protein tyrosine kinase	Pkinase_Tyr	80	81	82	78	79	82	85	93	99	91	178	244	222	268	0.0000
Protein-L-isoaspartate(D-aspartate) O-methyl	PCMT	2	7	6	1	1	3	3	4	3	3	18	17	13	21	0.0000
Proteins of unknown function (DUF2006)	DUF2006	0	0	5	0	0	0	0	0	0	0	0	1	0	1	0.0000
Putative ATPase subunit of terminase (gpP-lik	Terminase_5	0	0	0	0	0	0	0	0	0	0	3	0	0	1	0.0184
Putative esterase	Esterase	6	4	5	4	2	4	4	3	3	4	6	9	6	13	0.0000
Putative serine esterase (DUF676)	DUF676	7	4	3	2	1	2	2	2	1	1	10	7	8	12	0.0307
Pyridine nucleotide-disulphide oxidoreductas	Pyr_redox	30	41	59	21	20	25	21	27	28	30	65	53	42	66	0.0000
Pyridine nucleotide-disulphide oxidoreductas	Pyr_redox_2	69	87	127	40	35	59	51	53	54	52	90	82	69	99	0.0000
Pyridoxal-phosphate dependent enzyme	PALP	11	13	14	9	9	13	13	9	12	12	18	18	12	20	0.0184
Pyridoxamine 5'-phosphate oxidase	Pyridox_oxidase	4	6	3	3	1	4	3	4	4	3	6	3	7	5	0.0184
Rab5 binding	Rab5-bind	1	0	3	0	1	0	0	0	0	0	0	4	1	2	0.0000
Rad50 zinc hook motif	Rad50_zn_hook	2	0	0	0	1	0	0	0	0	0	1	3	0	1	0.0307
RecF/RecN/SMC N terminal domain	SMC_N	17	11	12	9	9	12	11	10	13	13	114	91	91	99	0.0000
Reovirus sigma C capsid protein	Reo_sigmaC	0	0	0	0	0	0	0	0	0	1	21	8	14	19	0.0000
Response regulator receiver domain	Response_reg	14	16	19	9	6	7	7	9	9	9	14	11	11	17	0.0000
Reverse transcriptase (RNA-dependent DNA pol	RVT_2	0	0	2	3	0	0	0	0	0	1	0	5	0	0	0.0000
Reverse transcriptase (RNA-dependent DNA pol	RVT_1	1	26	2	2	6	4	1	4	1	3	3	4	4	4	0.0000
Ribosomal RNA adenine dimethylase	RrnaAD	5	4	6	2	1	3	3	4	3	3	12	8	4	13	0.0000
RIM-binding protein of the cytomatrix active	Cast	0	1	0	0	0	2	2	0	1	1	0	0	3	0	0.0307
RIO1 family	RIO1	3	2	2	2	2	4	4	6	4	4	14	27	26	31	0.0000
RNA binding motif	RRM_3	1	0	2	0	1	1	0	2	0	0	3	6	3	7	0.0000
RNA polymerase III subunit RPC82	RNA_pol_Rpc82	1	1	1	1	1	1	1	1	1	1	3	1	0	4	0.0184

RNase H	RnaseH	3	19	1	3	2	3	4	2	2	2	2	2	2	4	0.0000
Roadblock/LC7 domain	Robl_LC7	1	2	2	1	0	1	1	0	1	1	2	0	0	1	0.0307
RTA1 like protein	RTA1	6	21	16	4	6	10	10	8	7	7	11	7	7	16	0.0000
S-antigen protein	S-antigen	0	0	0	0	0	0	0	0	0	0	2	0	1	4	0.0000
Saccharopine dehydrogenase	Saccharop_dh	15	19	23	3	5	8	8	9	8	9	17	21	15	19	0.0000
Secretory lipase	LIP	1	3	4	0	1	2	2	2	1	1	2	5	5	4	0.0450
Semialdehyde dehydrogenase, NAD binding doma	Semialdhyde_dh	3	4	11	4	5	3	3	3	4	4	10	7	8	8	0.0307
Sensors of blue-light using FAD	BLUF	0	0	0	0	0	0	0	0	1	1	0	2	2	0	0.0000
Septation ring formation regulator, EzrA	EzrA	0	0	0	0	0	0	0	0	0	0	1	3	0	2	0.0184
Septum formation initiator	DivIC	4	1	1	0	5	3	2	4	5	6	2	7	3	4	0.0000
Serine carboxypeptidase	Peptidase_S10	4	11	5	5	5	11	11	7	10	10	14	8	11	12	0.0000
Serine-threonine phosphatase 2A subunit B al	PP2A_B_N	5	1	1	2	1	3	0	1	1	1	17	12	13	9	0.0184
Shikimate / quinate 5-dehydrogenase	Shikimate_DH	13	16	16	5	4	5	7	7	5	4	26	29	20	30	0.0000
SHNi-TPR	SHNi-TPR	2	4	5	1	2	3	2	2	3	3	5	2	2	4	0.0184
short chain dehydrogenase	adh_short	68	125	165	50	44	78	59	54	68	68	116	118	85	125	0.0000
Sigma-70, region 4	Sigma70_r4	0	0	0	0	0	0	0	0	1	1	1	0	0	2	0.0307
SRR1	SRR1	0	1	0	2	1	1	1	1	1	1	0	1	0	2	0.0307
Subtilase family	Peptidase_S8	10	6	3	5	8	16	18	19	18	19	31	22	25	30	0.0000
Sugar (and other) transporter	Sugar_tr	77	145	181	54	50	75	71	62	70	70	95	78	83	88	0.0000
Sulfatase	Sulfatase	6	8	10	5	4	6	6	9	6	6	13	9	8	13	0.0184
Tannase and feruloyl esterase	Tannase	1	5	9	0	0	2	2	0	0	0	0	0	0	0	0.0307
TAP-like protein	Abhydrolase_4	1	1	3	0	0	2	2	2	0	0	3	1	4	1	0.0000
Taurine catabolism dioxygenase TauD, TfdA fa	TauD	13	14	21	3	2	4	4	5	5	5	6	5	6	6	0.0000
Tellurite resistance protein TehB	TehB	1	0	1	0	0	1	1	1	1	1	5	0	3	3	0.0000
Tetratricopeptide repeat	TPR_4	4	6	18	4	6	8	5	5	8	8	11	6	10	15	0.0000
Tetratricopeptide repeat	TPR_2	47	54	68	42	48	55	49	42	48	47	61	62	47	64	0.0000
Tetratricopeptide repeat	TPR_1	42	45	59	36	36	46	42	33	40	37	54	55	42	56	0.0000
Tetratricopeptide repeat	TPR_3	4	2	11	1	4	3	2	2	3	3	2	5	6	3	0.0000
TFIIE alpha subunit	TFIIE_alpha	1	1	1	1	1	1	1	1	1	1	6	2	1	5	0.0000
Thi4 family	Thi4	22	33	35	14	10	15	8	12	14	15	33	44	28	51	0.0000
Thiamine pyrophosphate enzyme, C-terminal TP	TPP_enzyme_C	5	7	7	5	5	6	6	6	7	7	18	11	9	20	0.0000
Thiamine pyrophosphate enzyme, central domai	TPP_enzyme_M	8	8	7	6	6	7	5	6	7	7	13	9	9	16	0.0000
Thiamine pyrophosphate enzyme, N-terminal TP	TPP_enzyme_N	5	6	6	5	5	5	5	4	6	6	12	6	6	13	0.0000
ThiF family	ThiF	9	9	8	8	9	8	8	8	8	7	14	9	8	14	0.0000
Thiolase, C-terminal domain	Thiolase_C	6	7	8	6	8	5	5	7	6	6	10	13	10	18	0.0000
Thiolase, N-terminal domain	Thiolase_N	9	14	21	9	6	18	11	9	12	12	31	29	17	36	0.0000
Transferase family	Transferase	1	6	7	1	0	4	1	2	1	1	5	4	3	2	0.0307
Transforming acidic coiled-coil-containing p	TACC	0	0	0	1	0	0	0	0	0	0	0	2	0	1	0.0184
Transketolase, C-terminal domain	Transketolase_C	4	3	6	4	3	4	3	3	3	3	5	10	4	11	0.0000
Transketolase, pyrimidine binding domain	Transket_pyr	4	5	6	4	4	4	4	4	4	4	5	12	5	13	0.0000
Transketolase, thiamine diphosphate binding	Transketolase_N	1	2	4	1	1	1	1	1	1	1	3	5	1	6	0.0000
Transmembrane protein	Macoilin	0	0	0	0	0	0	0	0	0	0	5	3	6	2	0.0184
Transposase	Transposase_8	1	16	1	1	0	0	0	0	0	0	2	1	0	2	0.0000
TrkA-N domain	TrkA_N	4	12	13	3	5	9	7	4	9	8	10	8	7	16	0.0000
tRNA methyl transferase	tRNA_Me_trans	1	1	0	1	0	1	1	0	0	0	5	4	1	5	0.0000
Tryptophan halogenase	Trp_halogenase	0	1	10	0	1	3	3	1	0	0	9	7	7	11	0.0000
Type I phosphodiesterase / nucleotide pyroph	Phosphodiest	6	7	10	7	8	8	7	9	8	8	13	9	7	13	0.0000
Type III restriction enzyme, res subunit	ResIII	21	23	20	22	19	22	23	19	19	19	79	62	70	84	0.0000
UbiA prenyltransferase family	UbiA	3	7	5	2	0	2	2	2	2	2	5	2	2	2	0.0184
UDP-glucose/GDP-mannose dehydrogenase family	UDPG_MGDP_dh_N	3	4	1	1	1	1	1	0	0	0	4	5	3	8	0.0000
Uncharacterised conserved protein (DUF2305)	DUF2305	3	2	1	1	1	1	2	1	1	1	8	5	8	4	0.0307
Vacuolar sorting protein 39 domain 1	Vps39_1	1	1	1	1	2	1	1	1	1	1	5	1	1	8	0.0000
Viral (Superfamily 1) RNA helicase	Viral_helicase1	2	2	2	3	2	3	3	2	6	6	27	16	22	25	0.0000
WD domain, G-beta repeat	WD40	111	116	121	110	113	116	113	110	110	109	161	126	146	152	0.0000
Xylose isomerase-like TIM barrel	AP_endonuc_2	6	7	7	3	4	2	2	4	4	5	4	6	3	8	0.0184
Zinc finger, C2H2 type	zf-C2H2	53	51	55	42	49	41	45	41	58	48	61	46	46	53	0.0184
Zinc finger, C3HC4 type (RING finger)	zf-C3HC4	36	35	34	29	29	31	34	30	38	37	54	38	48	48	0.0000
Zinc knuckle	zf-CCHC	11	7	9	23	9	8	7	7	10	17	7	8	8	9	0.0000
Zinc-binding dehydrogenase	ADH_zinc_N	40	70	90	33	29	36	33	35	33	34	70	58	55	75	0.0000

*Reported tree-wide p-values were corrected for multiple hypothesis testing using the Benjamini-Hochberg false discovery rate method.
