# Supplementary material for: Comparative Phylogenomics of Pathogenic and Nonpathogenic Species
Source: G3 (Bethesda). 2015 Nov 25;6(2):235–44. doi: 10.1534/g3.115.022806 (PMC4751544; doi:10.1534/g3.115.022806)
Supplement: Supporting Information [file supp_g3.115.022806_TableS2.pdf]

Table S2. Summary of genes showing evidence of positive selection by the Branch Site REL test (KOSAKOVSKY POND *et al.* 2011).

ID	Gene	p-value *	Expression **
CIMG_00040	Conserved hypothetical protein (DUF2235)	0.005451	-
CIMG_00251	Ulp1 protease family protein	0.013855	-
CIMG_00329	Nuclear cohesin complex subunit	0.015047	upH
CIMG_00547	RNA methyltransferase, TrmH family, group 3	0.036719	-
CIMG_00591	COP9 signalosome complex subunit 7a	0.002332	-
CIMG_00809	High-affinity nickel transporter (DNA polymerase lambda)	0.000027	-
CIMG_00815	High-affinity nickel transporter nic1	0.034231	upS
CIMG_01135	GINS DNA replication complex subunit Sld5	0.036659	-
CIMG_01175	Asparaginase	0.019608	-
CIMG_01214	Conserved hypothetical protein	0.010919	upS
CIMG_01394	Subtilisin-like protease	0.000976	-
CIMG_01420	Mechanosensitive ion channel family protein	0.000396	-
CIMG_01426	Gamma-butyrobetaine hydroxylase subfamily	0.014706	upH
CIMG_01507	Conserved hypothetical protein	0.006346	upH
CIMG_01625	Formamidopyrimidine-DNA glycosylase	0.000561	-
CIMG_01643	RNA methylase	0.004439	-
CIMG_01661	Conserved hypothetical protein (putative autophagy regulatory protein Atg2)	0.024921	upH
CIMG_01785	Conserved hypothetical protein	0.037151	-
CIMG_01987	Conserved hypothetical protein (DUF2467, C2H2 type zinc finger)	0.043760	upH
CIMG_02041	RNA binding protein Rnp24	0.031928	-
CIMG_02219	Conserved hypothetical protein (C2H2 type zinc finger)	0.010849	-
CIMG_02259	Conserved hypothetical protein (DUF2403, DUF2401)	0.000192	upH
CIMG_02333	Nucleolin	0.031243	-
CIMG_02376	Conserved hypothetical protein	0.000168	upS
CIMG_02498	Conserved hypothetical protein	0.014301	upS
CIMG_02797	Mitochondrial mRNA processing protein PET127	0.019366	upS
CIMG_02871	Phosphoglycerate mutase	0.033470	-
CIMG_02984	TCTP family protein	0.001607	-
CIMG_03196	TGF beta receptor associated protein 1	0.003769	-
CIMG_03224	dsDNA-dependent ATPase	0.013072	-
CIMG_03322	Conserved hypothetical protein (F-box domain)	0.016531	upH
CIMG_03340	DEAD box RNA helicase HelA	0.003518	-
CIMG_03363	Transcription initiation protein SPT3	0.049659	-
CIMG_03489	Aldose1-epimerase	0.002085	upH
CIMG_03508	Nucleolar protein NOP58	0.008107	-
CIMG_03719	Compass component spp1	0.013394	-
CIMG_03786	Conserved hypothetical protein	0.000020	upH
CIMG_03788	STE/STE11/BCK1 protein kinase	0.000629	-
CIMG_03998	Vacuolar protein sorting-associated protein 74	0.034655	upS
CIMG_04059	Endosomal cargo receptor	0.020312	-
CIMG_04060	SWR1-complex protein 4	0.028010	-
CIMG_04097	Conserved hypothetical protein (Tat pathway signal sequence)	0.010805	-
CIMG_04470	Membrane bound C2 domain-containing protein	0.002063	upH
CIMG_04486	Peptidyl-prolyl cis-trans isomerase	0.000194	-
CIMG_04533	Conserved hypothetical protein (MIZ/SP-RING zinc finger)	0.002411	-
CIMG_04547	Structure-specific endonuclease subunit SLX4	0.012354	-
CIMG_04822	DNA topoisomerase 1	0.034456	-
CIMG_04974	Conserved hypothetical protein (SRP40, C-terminal domain)	0.000005	-
CIMG_05122	Conserved hypothetical protein (PAPA-1-like conserved region)	0.000248	upS
CIMG_05165	Urease accessory protein UreG	0.019367	-
CIMG_05245	Thiamine pyrophosphokinase	0.020864	-
CIMG_05288	Conserved hypothetical protein	0.004166	upS
CIMG_05421	Beta-glucan synthesis-associated protein SKN1	0.017344	-
CIMG_05496	DNA polymerase epsilon subunit C	0.024757	-
CIMG_05542	Phosphoglycerate mutase	0.022010	upS
CIMG_05620	Conserved hypothetical protein	0.023260	upH
CIMG_05640	Isochorismatase family protein	0.004831	-
CIMG_05876	G1/S-specific cyclin Pcl5	0.001300	-
CIMG_05901	Proline permease	0.012280	-
CIMG_06294	Conserved hypothetical protein (Glutathione-dependent formaldehyde- activating enzyme)	0.013024	upS

CIMG_06361	Class V myosin	0.001249	-
CIMG_06502	Conserved hypothetical protein	0.007543	upH
CIMG_06553	Putative SH3 domain protein Cyk3	0.021649	upH
CIMG_06582	Phosphatidylinositol transfer protein SFH5	0.017966	upH
CIMG_06731	Conserved hypothetical protein	0.006763	-
CIMG_06955	Histone-lysine N-methyltransferase Clr4	0.046382	-
CIMG_06994	Cu-Zn superoxide dismutase	0.011593	-
CIMG_07095	NADH:ubiquinone oxidoreductase subunit	0.009568	-
CIMG_07110	Conserved hypothetical protein	0.026909	-
CIMG_07139	Protein phosphatase 2C	0.026973	-
CIMG_07513	Conserved hypothetical protein	0.029460	-
CIMG_07608	Conserved hypothetical protein	0.036740	upS
CIMG_07629	Leucine aminopeptidase 1	0.006461	-
CIMG_07728	Conserved hypothetical protein	0.012348	-
CIMG_07814	DNA-directed RNA polymerase II polypeptide	0.042025	-
CIMG_07846	Conserved hypothetical protein	0.004436	-
CIMG_08085	Conserved hypothetical protein	0.025896	upS
CIMG_08095	Conserved hypothetical protein	0.033971	-
CIMG_08300	Phospholipase B	0.035882	-
CIMG_08779	Conserved hypothetical protein (Putative methyltransferase)	0.022801	upS
CIMG_08791	Conserved hypothetical protein	0.000328	-
CIMG_08827	Conserved hypothetical protein	0.013534	upH
CIMG_08892	bZIP transcription factor	0.000129	upH
CIMG_08965	Conserved hypothetical protein (homeobox domain)	0.000013	-
CIMG_09023	Nuclear and cytoplasmic polyadenylated RNA-binding protein (pub1)	0.049048	upH
CIMG_09059	Serine palmitoyltransferase 2	0.000257	upS
CIMG_09408	Conserved hypothetical protein	0.020192	upS
CIMG_09449	ATPase synthesis protein 25	0.017249	-
CIMG_09551	60S ribosome biogenesis protein Mak11	0.036159	-
CIMG_09625	Conserved hypothetical protein (Transcription factor Pcc1)	0.033747	upH
CIMG_09696	Proline-rich antigen	0.011392	-
CIMG_09789	Conserved hypothetical protein (CS domain)	0.023226	-
CIMG_09801	1,3-beta-glucanosyltransferase	0.042396	-
CIMG_10180	Pheromone-dependent cell cycle arrest protein Far11	0.019899	upS
CIMG_10184	RING finger protein	0.036435	-
CIMG_10236	Mismatch-specific thymine-DNA glycosylase	0.019565	upS
CIMG_11422	C2H2 transcription factor	0.023987	upS
CIMG_11441	Ubiquitin-protein ligase	0.008829	-
CIMG_11666	Carboxypeptidase S1	0.014534	upH
CIMG_11667	Polymerase	0.034746	upH
CIMG_12068	Multicopper oxidase	0.028114	upS
CIMG_13167	Beta-glucosidase5	0.014984	upH
CIMG_13734	Glutaredoxin	0.003492	-

* Reported p-values were corrected for multiple hypothesis testing using the Bonferroni method.
** Previously reported expression differences (WHISTON *et al.* 2012) indicated at up-regulated in saprobic hyphae (upH), up-regulated in parasitic spherules (upS) or no reported expression difference (-).
